# Loss of the Asn333 high-mannose *N*-glycan impairs ACP3 folding and drives disease-associated dysfunction

**DOI:** 10.1016/j.isci.2026.117746

**Published:** 2026-10-07

**Authors:** Noriyoshi Manabe, Kazuyoshi Itoh, Shiho Ohno, Ayumi Miura, Aika Miyata, Sora Yashima, Sachiko Komatsu, Tsutomu Fujimura, Hisatoshi Hanamatsu, Jun-ichi Furukawa, Kiyohiko Angata, Akira Togayachi, Kiyoko F. Aoki-Kinoshita, Yoshihiro Akimoto, Tadashi Kaname, Shoko Nishihara, Yoshiki Yamaguchi

**Affiliations:** 1 Division of Structural Glycobiology, Institute of Molecular Biomembrane and Glycobiology, Tohoku Medical and Pharmaceutical University, Sendai, Miyagi 981-8558, Japan; 2 Glycan and Life Systems Integration Center (GaLSIC), Soka University, Hachioji, Tokyo 192-8577, Japan; 3 Division of Bioanalytical Chemistry, Tohoku Medical and Pharmaceutical University, Sendai, Miyagi 981-8558, Japan; 4 Institute for Glyco-Core Research (iGCORE), Nagoya University, Nagoya 466-8550, Japan; 5 Department of Microscopic Anatomy, Kyorin University School of Medicine, Mitaka, Tokyo 181-8611, Japan; 6 Department of Genome Medicine, National Center for Child Health and Development, Tokyo 157-8535, Japan

**Keywords:** ACP3, site-specific *N*-glycosylation, proteostasis, glycan-protein interactions, conformational stability, missense mutation, neurodevelopmental disease

## Abstract

Missense mutations disrupting *N*-glycosylation motifs can perturb protein folding and trafficking, yet the site-specific contributions of individual *N*-glycans to proteostasis remain poorly defined. Here, we delineated the functional hierarchy of three *N*-glycosylation sites in human prostatic acid phosphatase (ACP3). Glycoproteomic analysis showed that Asn94 and Asn220 carry predominantly complex-type *N*-glycans, whereas Asn333 is enriched in high-mannose structures. Glycan-deficient N94Q and N220Q variants were efficiently secreted and retained phosphatase activity, whereas N333Q and T335M accumulated intracellularly, formed endoplasmic reticulum aggregates, and lacked enzymatic activity. Molecular dynamics simulations revealed that the Asn333 high-mannose glycan forms persistent hydrogen-bond networks with surrounding residues and exhibits markedly reduced conformational flexibility, supporting a non-redundant structural role. Individuals harboring a heterozygous p.T335M variant presented severe neurodevelopmental abnormalities, linking Asn333 glycan loss to proteostasis failure *in vivo*. These findings establish a site-specific glycan-protein stabilizing mechanism with broad implications in glycoprotein folding and human disease.

## Highlights

• Site-specific glycoproteomics identifies Asn333 as a high-mannose *N*-glycosylation site
• The Asn333 *N*-glycan is required for efficient ER exit and secretion of ACP3
• Molecular dynamics simulations reveal structural stabilization by the Asn333 *N*-glycan
• Disease-associated T335M abolishes Asn333 glycosylation and impairs ACP3 folding

## Graphical abstract

**Figure:**
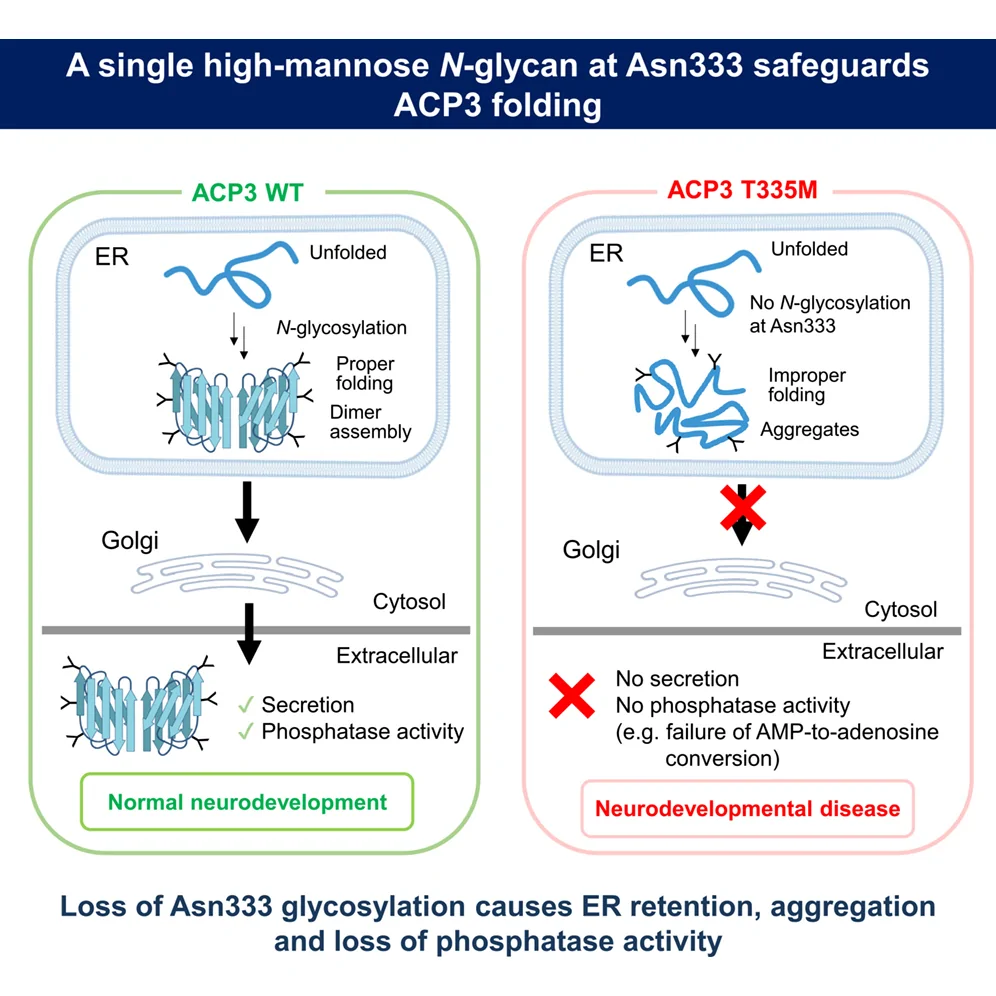

## Teaser

Neuroscience; Molecular neuroscience; Developmental neuroscience; Cellular neuroscience; Protein structure aspects; Protein folding

## Introduction

Protein *N*-glycosylation is a site-selective yet structurally heterogeneous post-translational modification that reshapes protein folding, affecting conformational stability, intracellular trafficking, and cellular proteostasis. *N*-glycans are added co-translationally at asparagine residues within the consensus sequon Asn-X-Ser/Thr (X ≠ Pro) in the endoplasmic reticulum (ER), where they participate in quality-control pathways and influence nascent polypeptide folding. They are broadly classified into high-mannose, hybrid, and complex types according to the extent of Golgi-mediated processing. High-mannose *N*-glycans, also referred to as oligomannose-type, are defined as mannose_5–9_*N*-acetylglucosamine_2_ (Man_5–9_GlcNAc_2_) structures that escape extensive Golgi trimming and retain mannose-rich outer arms. Although *N*-glycosylation broadly facilitates protein maturation, increasing evidence suggests that individual glycosylation sites can exert distinct and non-equivalent structural effects. Consistent with this notion, pathogenic missense variants that abolish or create *N*-glycosylation sequons have been associated with multiple inherited disorders.^1^^,^^2^^,^^3^^,^^4^ However, the molecular principles by which specific *N*-glycans stabilize protein architecture, maintain proteostasis, and determine functional output remain incompletely defined.

Prostatic acid phosphatase (PAP; ACP3) is a secreted *N*-glycoprotein whose folding, dimerization, and enzymatic activity are tightly coupled to its maturation in the secretory pathway. It is a homodimeric acid phosphatase that catalyzes dephosphorylation of diverse substrates under acidic conditions (pH 4–6),^5^^,^^6^^,^^7^^,^^8^^,^^9^ and its secretion is essential for functional activity. Although ACP3 is best known for its high expression in the prostate and historical use as a prostate cancer biomarker,^10^ it is also expressed in several non-prostatic tissues, including the central nervous system.^11^^,^^12^ Notably, ACP3 transcripts and protein have been detected in brain regions implicated in language processing, motor coordination, and higher cognitive functions.^11^^,^^12^ These observations suggest that ACP3 proteostasis may extend beyond prostate physiology; however, the structural determinants that govern its maturation, stability, secretion, and their potential relevance to neurological phenotypes remain largely undefined.

Structurally, mature ACP3 assembles into a stable 100-kDa homodimer, and dimerization is required for phosphatase activity.^13^ Crystal and cryo-electron microscopy (cryo-EM) structures of glycosylated human ACP3 revealed three *N*-glycosylation sites per polypeptide chain,^14^^,^^15^ while NMR and mass spectrometric analyses defined the glycan structures as a complex-type *N*-glycan at Asn94 and Asn220 and a high-mannose-type *N*-glycan at Asn333.^16^^,^^17^^,^^18^ Due to its proximity to the catalytic regions, the Asn333 *N-*glycan has been proposed to modulate enzymatic function.^14^ Consistent with this idea, early studies using *Pichia pastoris* expression systems demonstrated that glycosylation at Asn333 is required for efficient secretion and enzymatic activity.^19^ Despite these observations, it remains unclear whether this site plays a structurally non-redundant role in maintaining ACP3 conformational integrity and proteostasis, or if disruption of this glycan contributes to human disease.

In this study, we have systematically investigated the functional hierarchy of ACP3 *N*-glycosylation sites by generating recombinant wild-type (WT) ACP3 and five glycan-deficient variants, and quantitatively assessing their secretion, enzymatic activity, intracellular localization, and structural stability. Integrating biochemical analyses with molecular dynamics (MD) simulations has enabled us to define a site-specific stabilizing mechanism centered on the Asn333 glycan. Finally, by examining clinical phenotypes associated with the ACP3 missense variant T335M, in which the Asn333 glycosylation sequon is disrupted, we can connect glycan loss to proteostasis failure and neurodevelopmental disease. Taken together, our study establishes a mechanistic framework in which a structurally non-redundant *N*-glycan safeguards ACP3 folding and secretion, thereby linking site-specific glycosylation to human disease.

## Results and discussion

### Asn333 *N*-glycosylation is essential for ACP3 secretion

Through an in-house database containing information on missense variants among patients with undiagnosed disease, we identified a heterozygous p.T335M variant in prostatic acid phosphatase (ACP3). Because Thr335 forms part of the canonical Asn333-X-Thr sequon in ACP3, this substitution is predicted to abolish *N*-glycosylation at Asn333. As the functional consequences of this variant have not previously been characterized, the clinical observations prompted us to systematically dissect the relative contribution of each *N*-glycosylation site to ACP3 folding, trafficking, and function.

Human ACP3 contains three *N*-glycosylation sites per polypeptide chain: Asn94, Asn220, and Asn333 (Figure 1A). All these sequons are evolutionarily conserved among mammalian orthologs, including mouse, rat, and bovine ACP3, suggesting the functional relevance of these sites. To dissect the contribution of each glycan, we generated individual glycosylation-deficient mutants (N94Q, N220Q, and N333Q), the disease-associated T335M variant, and a double mutant (N94Q/N220Q), alongside the WT recombinant protein (Figure 1B). All expression constructs were engineered with an N-terminal secretion signal and a C-terminal His_8_ tag to enable secretion and affinity purification.

**Figure 1:**
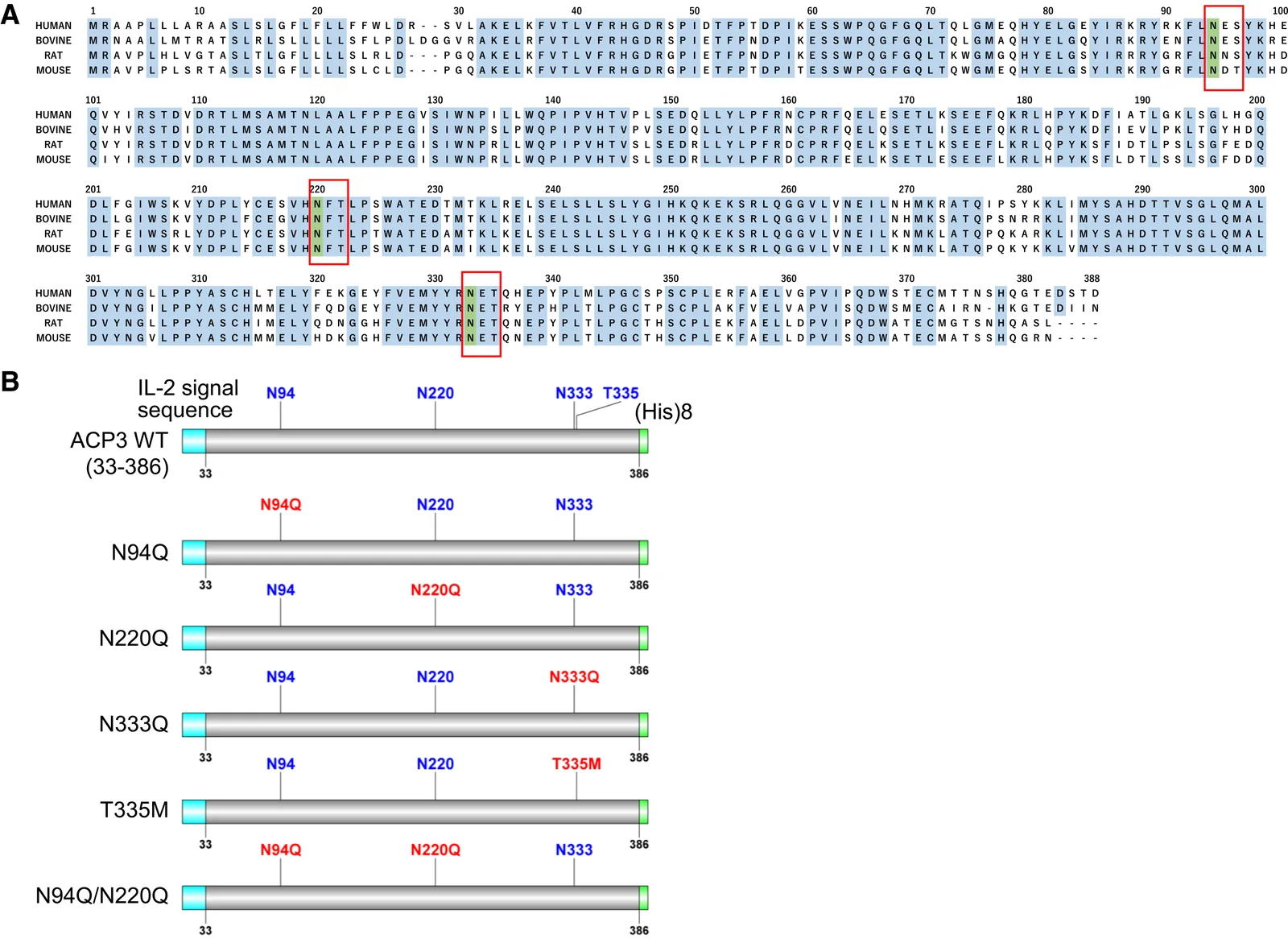
Evolutionary conservation of ACP3 *N*-glycosylation sites and design of glycan-deficient constructs (A) Multiple sequence alignment of ACP3 from human, mouse, rat, and bovine species. Conserved *N*-glycosylation sequons (Asn-X-Ser/Thr) are highlighted in red. The three *N*-glycosylation sites (Asn94, Asn220, and Asn333; human ACP3 numbering) are evolutionarily conserved among mammals. (B) Representation of the recombinant ACP3 constructs used in this study, showing the expressed region corresponding to residues 33–386 of human ACP3. *N*-glycosylation-deficient mutants (N94Q, N220Q, N333Q, and T335M) were generated to investigate the role of individual glycans. A double mutant (N94Q/N220Q) was generated to enable selective analysis of *N*-glycan structures at Asn333. The IL-2 secretion signal is shown in cyan, the ACP3 polypeptide in gray, and the C-terminal His_8_ tag in green.

To evaluate the impact of glycosylation on ACP3 secretion, culture supernatants from transfected cells were subjected to Ni^2+^-affinity purification. WT ACP3 was efficiently recovered from the supernatant (4.1 mg per 30 mL of culture). The N94Q and N220Q variants were also secreted, albeit at reduced levels (2.4 mg and 0.8 mg, respectively). In contrast, neither N333Q nor T335M was detected in the supernatant fractions (Figure 2A), indicating a severe secretion defect.

**Figure 2:**
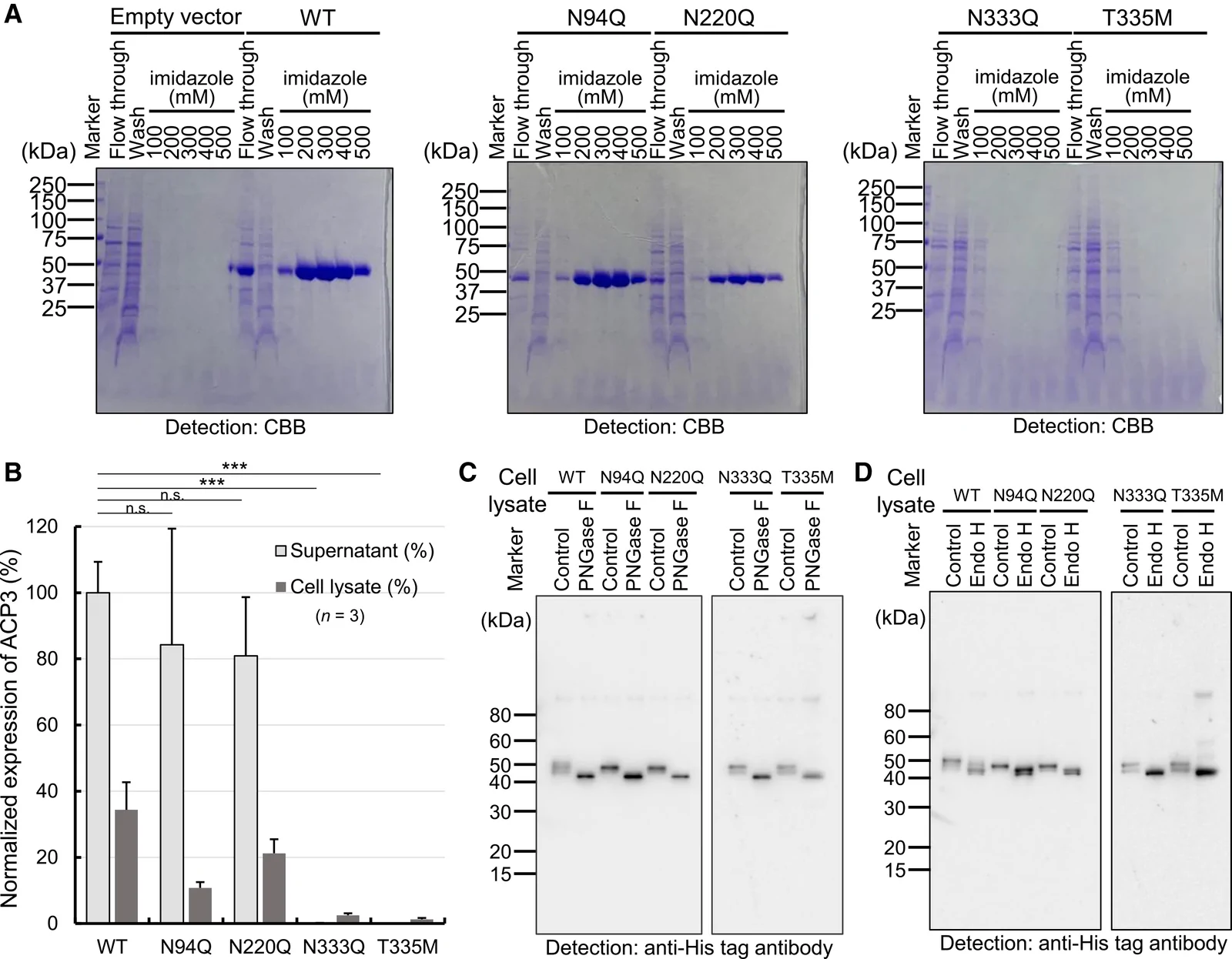
Loss of Asn333 glycosylation impairs ACP3 secretion and results in intracellular accumulation of immature glycoforms (A) Ni^2+^-affinity purification of His_8_-tagged ACP3 proteins from culture supernatants. Expi293F cells were transfected with empty vector (control) or constructs encoding WT, N94Q, N220Q, N333Q, or T335M ACP3. Eluted fractions obtained with increasing concentrations of imidazole (100–500 mM) were analyzed by SDS-PAGE. WT, N94Q, and N220Q proteins were recovered from the supernatant; N333Q and T335M were not detected. (B) Quantitative analysis of intracellular and secreted ACP3 variants in cells collected 4 days after transfection with expression plasmid. Intracellular ACP3 levels were normalized to the corresponding GAPDH signals. Transfection efficiency was monitored by measuring *Renilla* luciferase activity from a co-transfected pRL-TK plasmid, and sample loading for SDS-PAGE was adjusted accordingly. The amounts of intracellular and secreted ACP3 were quantified by densitometric analysis using Image Lab software (ver 6.1, Bio-Rad, CA, USA). Values are expressed relative to the amount of secreted WT ACP3 (defined as 100%). Data represent the mean ± SD (*n* = 3 independent experiments). Representative immunoblots of culture supernatants and corresponding cell lysates are shown in Figure S1. Statistical significance versus WT (supernatant) was assessed by Dunnett’s multiple-comparisons test (∗∗∗*p* < 0.001; n.s., not significant) using GraphPad Prism (ver. 9.5.1, GraphPad Software, MA, USA). (C) PNGase F treatment of intracellular ACP3. M-PER-solubilized cell lysates prepared 2 days after transfection were treated with PNGase F and analyzed by SDS-PAGE followed by immunoblotting with anti-His tag antibody. PNGase F treatment resulted in a single immunoreactive band, confirming that intracellular ACP3 is *N*-glycosylated. (D) Endo H treatment of intracellular ACP3. M-PER-solubilized cell lysates prepared 2 days after transfection were treated with Endo H and analyzed by SDS-PAGE followed by immunoblotting with anti-His tag antibody. Endo H treatment resulted in a marked mobility shift of the N333Q and T335M variants, indicating that these intracellularly retained proteins predominantly carry immature Endo H-sensitive *N*-glycans. WT ACP3, N94Q, and N220Q also exhibited Endo H sensitivity, consistent with the presence of a fraction of immature glycoforms that have not yet undergone complete Golgi processing.

To determine whether the latter two variants were retained intracellularly, both culture supernatants and corresponding cell lysates were analyzed by western blotting using anti-histidine tag antibody (Figure S1). To account for differences in transfection efficiency, *Renilla* luciferase activity from a co-transfected reporter plasmid was quantified in parallel and used to adjust sample loading. Intracellular ACP3 signals were normalized to the corresponding GAPDH signals to correct for differences in cell number. The amounts of intracellular and secreted ACP3 were then quantified by densitometry, and reported relative to that of secreted WT ACP3, defined as 100% (Figure 2B). This quantitative analysis confirmed that N94Q and N220Q ACP3 were secreted at 84% and 81%, respectively, of the WT level, whereas N333Q and T335M ACP3 were detected predominantly in the intracellular fractions, with only trace amounts present in the culture supernatants (0.14% and 0.05% of WT, respectively), indicating that these variants are largely retained in the ER.

To evaluate the oligomeric state of secreted ACP3, purified WT, N94Q, and N220Q proteins were analyzed by size-exclusion chromatography. All three proteins eluted as a single major peak with an apparent molecular mass consistent with a dimer, indicating that disruption of the Asn94 or Asn220 glycosylation site does not appreciably affect the oligomeric state of secreted ACP3 (Figure S2).

To characterize the *N*-glycosylation status of intracellular ACP3, Mammalian Protein Extraction Reagent (M-PER)-solubilized cell lysates were treated with PNGase F prior to immunoblot analysis. PNGase F treatment resulted in a single immunoreactive band, confirming that intracellular ACP3 is *N*-glycosylated (Figure 2C). In addition, Endo H treatment induced clear mobility shifts in intracellular WT ACP3, as well as in all of the ACP3 variants examined (Figure 2D), indicating the presence of Endo H-sensitive immature *N*-glycans. Notably, the N333Q and T335M variants also exhibited Endo H-sensitive mobility shifts into a single band, despite disruption of the N333 glycosylation sequon. These findings indicate that the remaining *N*-glycosylation sites are occupied predominantly with Endo H-sensitive immature *N*-glycans, consistent with impaired glycan maturation and retention within the early secretory pathway.

Collectively, these data demonstrate that *N*-glycosylation at Asn333 plays a crucial role in efficient ACP3 secretion, probably by promoting proper folding and facilitating escape from intracellular quality-control surveillance. In contrast, glycosylation at Asn94 seems largely dispensable for both secretion and oligomerization, whereas glycosylation at Asn220 contributes to efficient secretion without affecting the dimeric assembly of the secreted protein.

### Asn333 predominantly carries high-mannose *N*-glycans

To determine the site-specific nature of the immature glycans identified earlier, we performed focused protein glycomic analysis of WT and an N94Q/N220Q double mutant, in which only Asn333 remains glycosylated. In WT ACP3, hybrid- and complex-type (Hyb/Com) glycans accounted for approximately 60% of total *N*-glycans, whereas paucimannose- and high-mannose-type glycans represented the remaining 40% (Figures S3 and S4). In contrast, the glycan profile of the N94Q/N220Q mutant was markedly shifted toward high-mannose-type structures, with Hyb/Com glycans reduced to approximately 15%. Because the N94Q/N220Q construct preserves only the Asn333 sequon, the reduced abundance of Hyb/Com glycans in the double mutant is consistent with previous reports identifying Asn94 and Asn220 in ACP3 as major carriers of processed complex-type *N*-glycans.^16^^,^^17^^,^^18^

To validate this observation at the glycopeptide level, we next performed liquid chromatography-mass spectrometry (LC-MS) and liquid chromatography-tandem mass spectrometry (LC-MS/MS) analysis of trypsin-digested WT ACP3 to assign glycan structures in a site-specific manner (Figures 3A and S5). At Asn94, glycosylation of the corresponding peptide was 88%, with 12% in unglycosylated form (Figures 3C and S6). The glycans attached at Asn94 were predominantly complex-type structures (81%), and only a minority corresponded to high-mannose-type structures (7%). The most abundant composition was HexNAc_5_Hex_3_ (33%), consistent with either tri-antennary complex-type glycans or bi-antennary complex-type glycans containing a bisecting GlcNAc residue.

**Figure 3:**
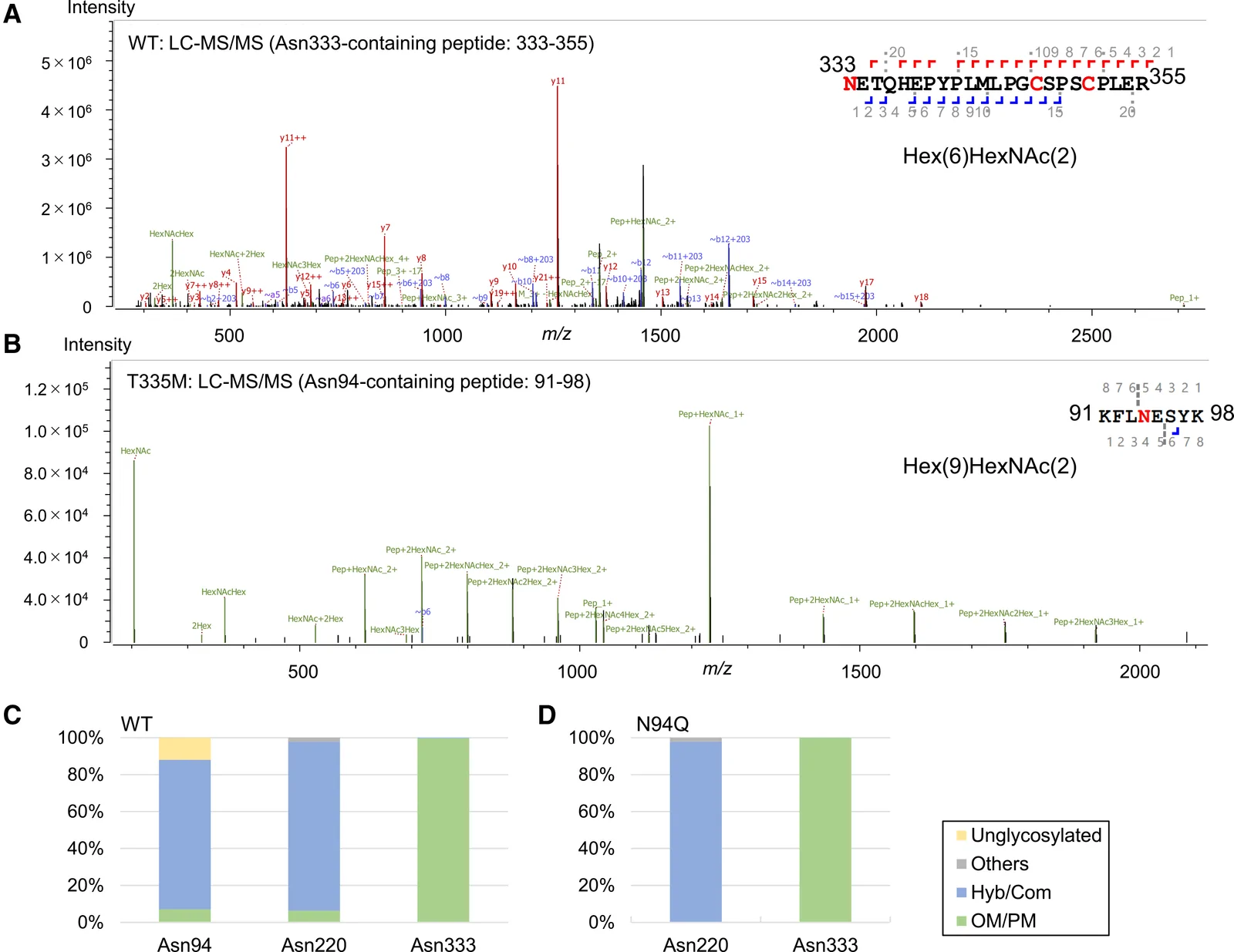
Site-specific *N*-glycan profiling reveals a high-mannose glycan at Asn333 (A) Representative LC-MS/MS spectrum of tryptic glycopeptide containing Asn333. Diagnostic fragment ions and glycan compositions were used to assign site-specific *N*-glycan structures. Representative LC-MS/MS spectra of glycopeptides containing Asn94 or Asn220 are shown in Figure S5. (B) Representative LC-MS/MS spectrum of an Asn94-containing glycopeptide identified from the intracellular T335M ACP3 variant, demonstrating the presence of a high-mannose glycoform (Man_9_GlcNAc_2_). (C) Quantitative comparison of the *N*-glycan composition at Asn94, Asn220, and Asn333 in WT ACP3. Relative abundances of hybrid/complex-type (Hyb/Com) and paucimannose/high-mannose-type (PM/HM) glycans were calculated from integrated glycopeptide intensities in LC-MS. (D) Quantitative comparison of the *N*-glycan composition at Asn220 and Asn333 in ACP3 N94Q. LC-MS analysis was also performed for ACP3 N220Q; however, glycopeptides were not detected with sufficient sensitivity to allow reliable site-specific glycan analysis (data not shown).

Asn220 showed almost full glycosylation (>99%). Similar to Asn94, complex-type glycans were dominant (91%) with HexNAc_4_Hex_3_ representing the most abundant composition (23%), while high-mannose-type glycans were detected at low abundance (6%). In striking contrast, no complex-type glycans were detected at Asn333. Instead, this residue was exclusively modified by high-mannose-type glycans (>99%), with Man_6_GlcNAc_2_ accounting for 78% of the total glycoforms observed.

In addition to WT, we analyzed the N94Q variant by site-specific LC-MS/MS. As expected, Asn220 was occupied predominantly by hybrid- and complex-type glycans, whereas Asn333 was enriched in high-mannose-type glycans (Figures 3D and S7), confirming that Asn333 is the principal carrier of immature high-mannose glycans in ACP3. Site-specific LC-MS/MS analysis was also performed for the N220Q variant; however, the glycopeptide signals were of insufficient intensity to allow confident glycopeptide identification and site-specific glycan assignment. Therefore, no further interpretation was made based on these data.

To further investigate the glycosylation status of intracellularly retained ACP3, LC-MS/MS analysis of intracellular N333Q and T335M was performed. Although only a limited number of glycopeptides could be identified due to the low abundance of intracellular ACP3, a glycopeptide carrying a high-mannose glycoform (Man_9_GlcNAc_2_) at Asn94 was detected in both variants (Figures 3B and S8), demonstrating that the remaining *N*-glycosylation sites in the intracellular N333Q and T335M proteins retain immature high-mannose-type glycans. These findings are consistent with the Endo H sensitivity of both variants (Figure 2D) and provide independent evidence that N333Q and T335M are retained in the ER before Golgi-mediated *N*-glycan maturation.

It should be noted that terminal galactose and especially sialic acid residues may be underrepresented under the present analytical conditions. In particular, sialic acid residues are known to be labile during glycan and glycopeptide MS analyses and might not be detected.^20^^,^^21^^,^^22^ Therefore, the reported glycan compositions primarily reflect the major glycan classes and core structural features at each glycosylation site rather than providing a complete representation of terminal glycan modifications.

Collectively, these data demonstrate clear site-specific differences in *N*-glycan processing within ACP3. Whereas Asn94 and Asn220 undergo extensive Golgi-mediated maturation to complex-type glycans, Asn333 remains predominantly in a high-mannose state. These findings are consistent with our MALDI-TOF-MS glycomic analysis (Figures S3 and S4) and previous reports.^16^^,^^17^^,^^18^

### ACP3 N333Q and T335M variants accumulate in the ER and form intracellular aggregates

To assess whether loss of site-specific *N*-glycosylation affects ACP3 intracellular trafficking, we conducted immunostaining of HEK293 cells transiently expressing the WT or variant ACP3 proteins. Whereas the WT, N94Q, and N220Q proteins predominantly colocalized with the *cis*-Golgi body marker GOLPH2 (Figure 4A, closed arrowheads and Video S1 and Figure S21), the N333Q and T335M proteins formed perinuclear aggregates that colocalized with the ER marker calnexin (Figure 4A, open arrowhead; and Videos S2 and S3, and Figures S22 and S23). In addition, nuclear deformation was observed in regions adjacent to the aggregates.

**Figure 4:**
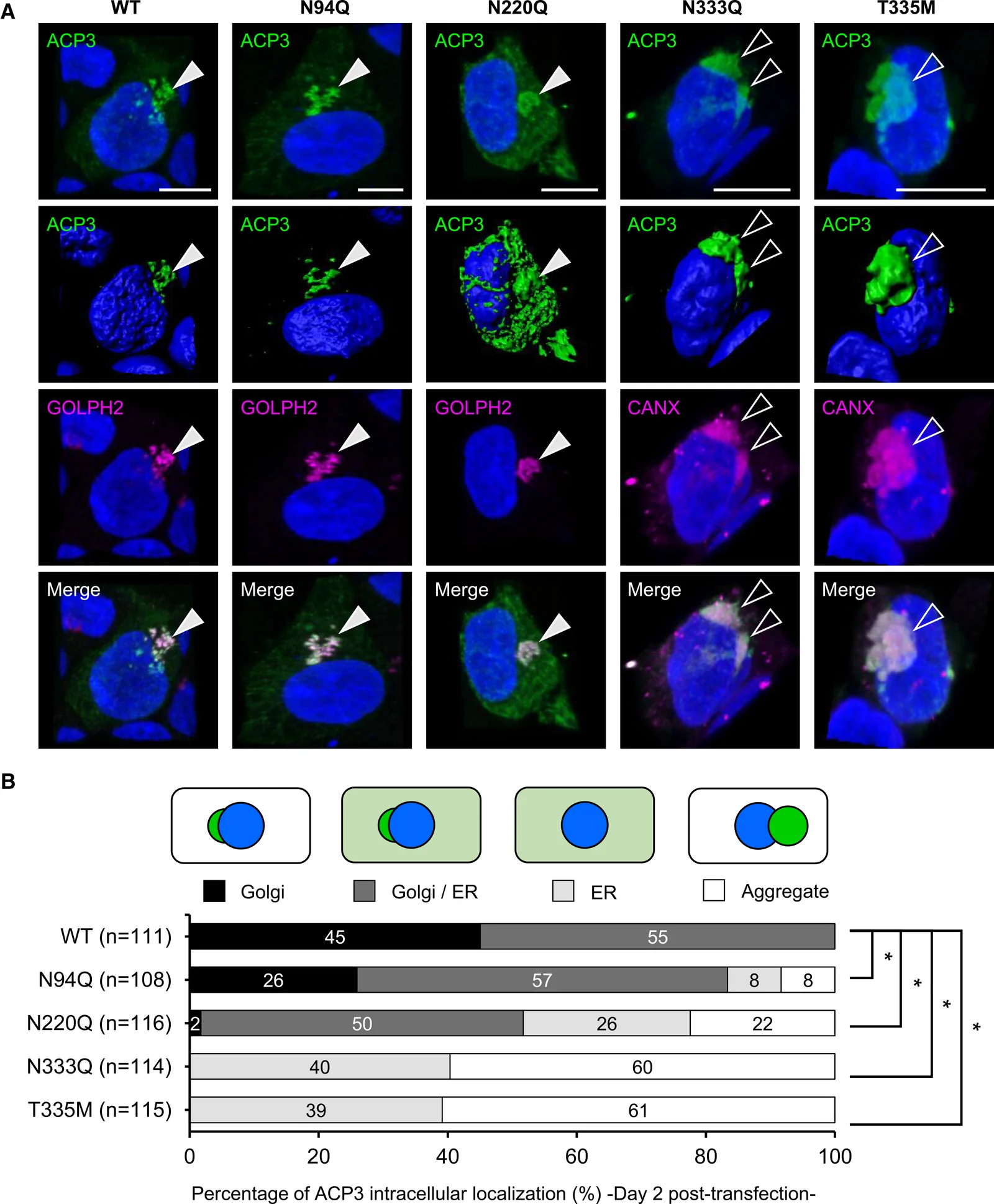
Loss of Asn333 glycosylation causes ER retention and intracellular aggregation of ACP3 (A) Representative 3D confocal images of HEK293 cells expressing WT or variant ACP3 (N94Q, N220Q, N333Q, and T335M) proteins. Surface-rendered models are shown in the second row. ACP3 and nuclei were labeled with anti-His tag antibody (green) and Hoechst 33258 (blue), respectively. Anti-GOLPH2 (magenta) and anti-calnexin (CANX, magenta) antibodies were used as markers for the *cis*-Golgi and ER, respectively. Closed arrowheads indicate WT, N94Q, and N220Q proteins colocalizing with GOLPH2. Open arrowheads indicate aggregates of N333Q and T335M proteins colocalizing with CANX. Scale bars, 10 μm. (B) Quantification of the intracellular localization of each ACP3 protein on day 2 after transfection. Localization was classified as Golgi, Golgi/ER, ER, or aggregate, as indicated by the shading. Statistical significance was evaluated by χ^2^ test (*n* = number of analyzed cells; ∗*p* < 0.0001).

Video S1. Three-dimensional confocal microscopy reconstruction of wild-type ACP3 localizing to the Golgi apparatus in HEK293 cells

Video S2. Three-dimensional confocal microscopy reconstruction of ACP3 N333Q forming aggregates within the endoplasmic reticulum in HEK293 cells

Video S3. Three-dimensional confocal microscopy reconstruction of ACP3 T335M forming aggregates within the endoplasmic reticulum in HEK293 cells

For a more quantitative analysis, the location of ACP3 in more than 100 cells was analyzed and characterized as Golgi, Golgi/ER, ER, or aggregate (Figure 4B). On day 2 after transfection, most cells expressing WT or N94Q showed Golgi or Golgi/ER localization of the protein (Figure 4B), although a few N94Q-expressing cells showed ER localization or aggregate formation. In cells expressing N220Q, the protein was roughly equally distributed between Golgi/ER and ER/aggregate. In contrast, the N333Q and T335M proteins showed only ER localization or aggregate formation, suggesting that ER exit was impaired due to misfolding. This observation is consistent with our aforementioned data showing that the N333Q and T335M variants were not secreted.

To examine whether ER structure is affected in the presence of aggregates, we analyzed ER morphology by confocal microscopy. Outside the aggregate-containing regions, ER structure remained intact in N333Q- and T335M-expressing cells (Figure S9), suggesting that aggregates do not overtly disrupt ER organization at the light microscopic level.

### Intraluminal ACP3 aggregates and ER lumen expansion in cells expressing N333Q and T335M variants

To further define the ultrastructural features of the aggregate-like structures observed by confocal microscopy, we performed transmission electron microscopy (TEM), which revealed the presence of electron-dense aggregates adjacent to deformed nuclei in cells expressing the T335M or N333Q protein (Figures 5A–5Bʹ), consistent with our confocal observations. The aggregates were covered by a membrane (Figures 5Aʹ and 5Bʹ), suggesting that they were localized within the ER lumen, as supported by their colocalization with ER markers (Figures 4A, S9, S22, and S23 and Videos S2 and S3). Furthermore, cells expressing N333Q or T335M protein showed enlarged ER lumens as compared with non-transfected and WT-expressing cells, while the Golgi structure remained unchanged (Figures 5C–5F), consistent with ER stress-like morphological alterations.

**Figure 5:**
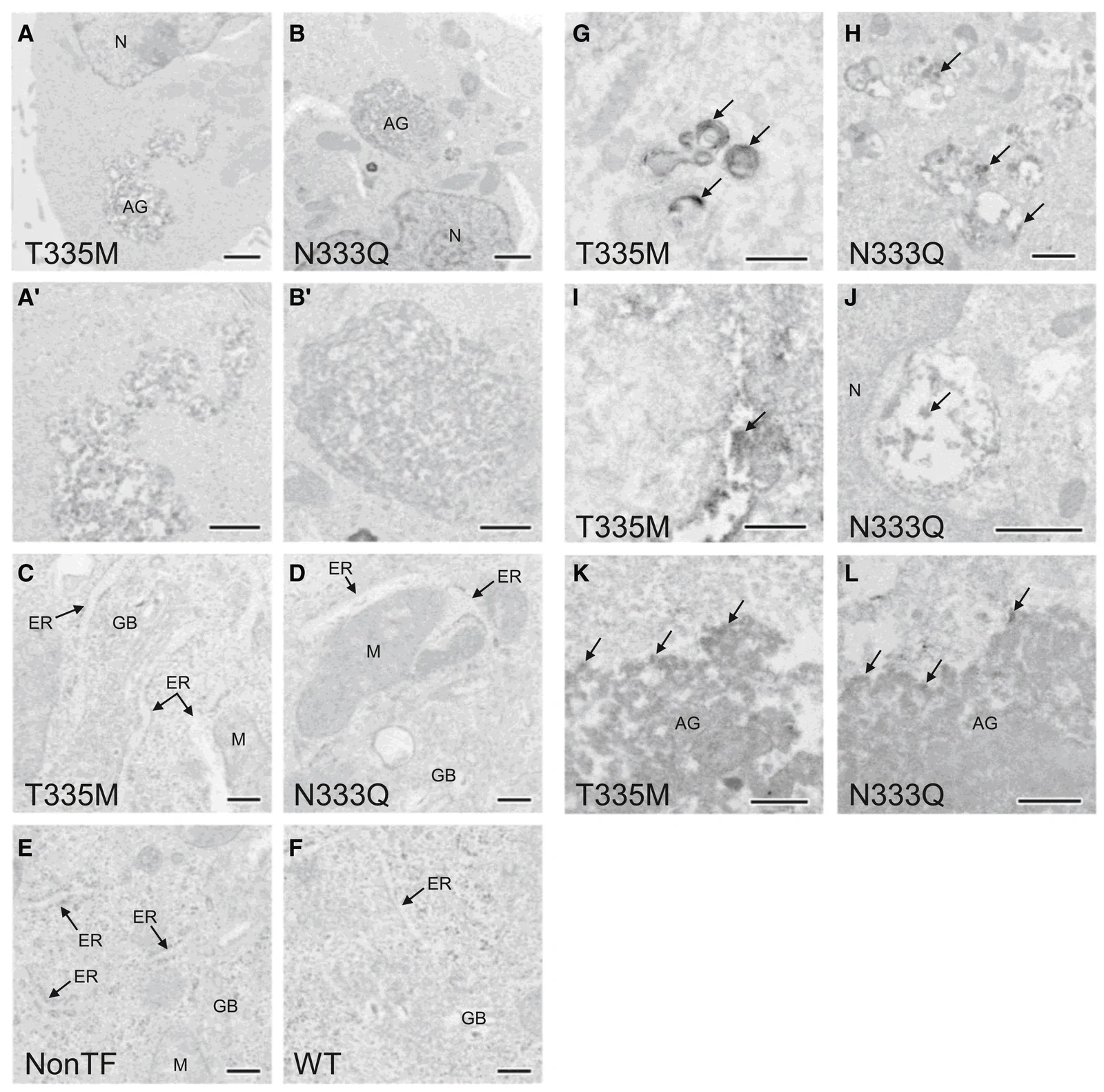
Ultrastructural evidence of ER expansion and membrane-bound aggregates in Asn333 glycan-deficient cells (A–F) Transmission electron micrographs of HEK293 cells expressing T335M (A, Aʹ, and C), N333Q (B, Bʹ, and D), or WT (F) ACP3 protein and non-transfected (NonTF) cells (E). Electron-dense aggregates (AGs), surrounded by a membrane, are visible in A and B, and shown at higher magnification in Aʹ and Bʹ, respectively. The lumen of the ER is expanded in cells expressing T335M (C) and N333Q (D) as compared with NonTF (E) and WT-expressing (F) cells. The Golgi body (GB) shows no significant changes (C–F). N, nucleus; M, mitochondria. (G–L) Immunoelectron micrographs showing localization of ACP3 in cells expressing T335M (G, I, and K) or N333Q (H, J, and L) ACP3 protein. Anti-His tag immunoreactivity (arrows) is observed in intracellular vesicles (G and H), within the ER (I and J), and at the periphery of aggregates (K and L). Scale bars: 1 μm (A and B), 500 nm (A′, B′, G–L), and 200 nm (C–F).

Immunoelectron microscopy of T335M- and N333Q-expressing cells using anti-His tag antibody further showed immunolabeling in intracellular vesicles, the expanded ER lumen, and the periphery of the aggregates (Figures 5G–5L, arrows). Immunolabeling was largely absent from the interior of aggregates, possibly due to limited antibody penetration.

Taken together, these findings demonstrate that loss of Asn333 glycosylation results in intraluminal accumulation and aggregation of ACP3 within the ER, accompanied by expansion of the ER lumen, thereby providing ultrastructural evidence of defective folding and ER retention of the variant proteins. Further studies will be required to determine whether these alterations activate either canonical ER stress or an unfolded protein response pathway.^23^^,^^24^^,^^25^^,^^26^

### Intracellular N333Q and T335M ACP3 variants exhibit increased protease susceptibility consistent with misfolding

Next, to examine whether the intracellular accumulation of N333Q and T335M reflects defective folding, we performed proteinase K sensitivity assays. Attempts to purify WT and glycan-deficient ACP3 variants from cell lysates by Ni^2+^-affinity chromatography resulted in markedly reduced recovery and co-elution of multiple cellular proteins, especially for cells expressing N220Q, N333Q, and T335M (Figure S10). Among the cellular proteins, an ER-resident lectin chaperone, calreticulin, was consistently detected in fractions of N220Q and T335M as representative glycan-deficient variants (Figure S11). The presence of calreticulin suggests that there may be an association between glycan-deficient ACP3 variants and ER quality-control components, consistent with impaired folding and retention in the ER. In line with this, subsequent protease sensitivity assays were performed using cell lysates expressing the WT or variant ACP3 proteins.

Equal amounts of total cell lysates were incubated with increasing concentrations of proteinase K, followed by immunoblot detection (Figure S12). Whereas ACP3 WT remained largely resistant to proteolytic digestion under these conditions, both the N333Q and T335M variants exhibited markedly increased susceptibility to proteinase K-mediated degradation (Figure 6A). N220Q showed a modest increase in protease sensitivity as compared with WT, albeit to a lesser extent than N333Q and T335M. The accelerated loss of immunoreactive signal in N333Q and T335M suggested reduced structural stability and increased exposure of protease-accessible regions in these proteins.

**Figure 6:**
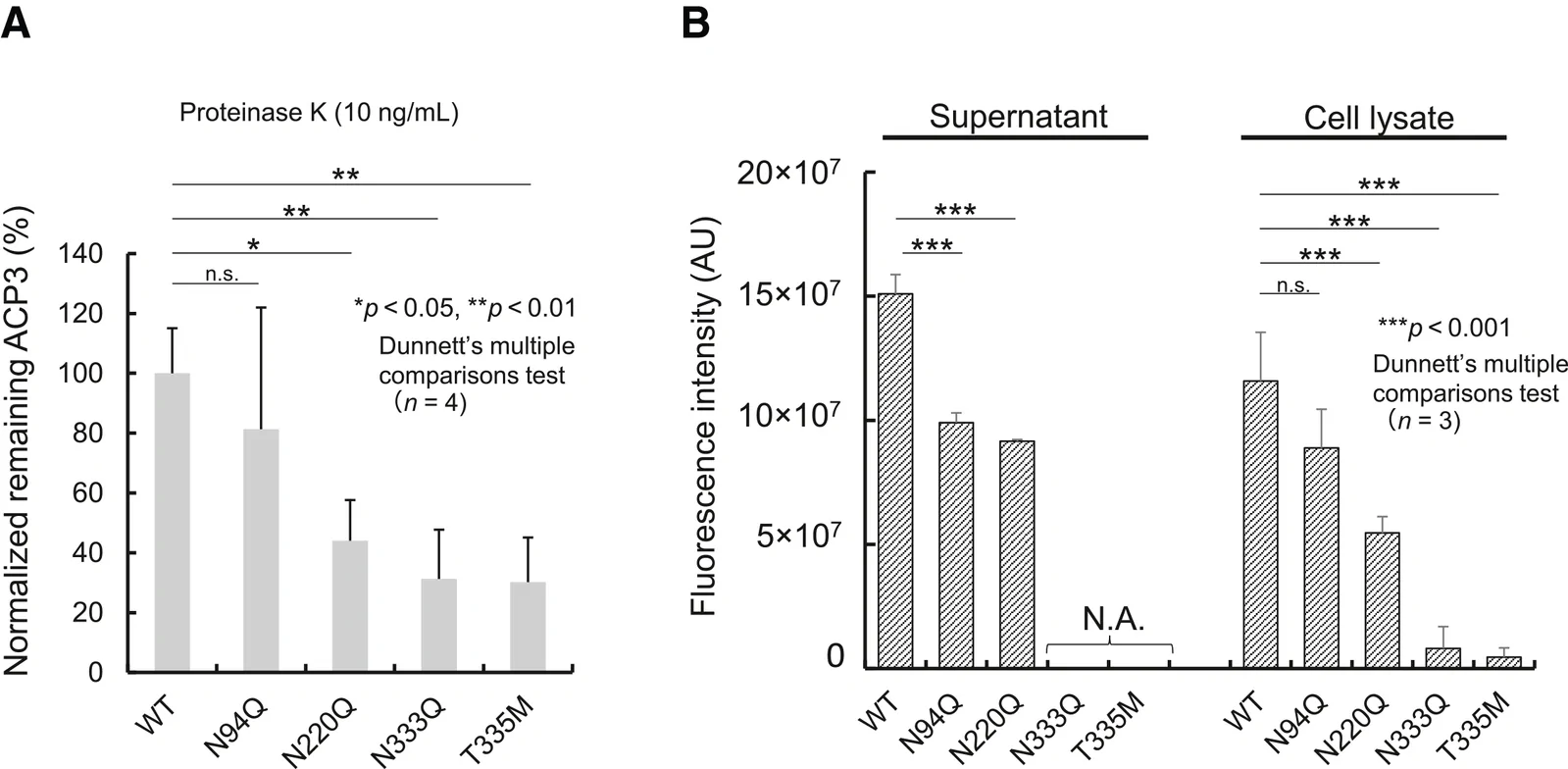
Loss of Asn333 glycosylation destabilizes ACP3 and abolishes phosphatase activity (A) Proteinase K sensitivity assay of ACP3 variant proteins. Cell lysates expressing WT or mutant ACP3 were incubated with 10 ng/mL of proteinase K at 37°C for 1 h, and the remaining ACP3 was quantified by immunoblotting. Band intensities were normalized to samples incubated without proteinase K (set to 100%). Data represent the mean ± SD from *n* = 4 independent experiments. Statistical significance versus WT was assessed by Dunnett’s multiple-comparisons test (∗*p* < 0.05, ∗∗*p* < 0.01; n.s., not significant) using GraphPad Prism (ver. 9.5.1, GraphPad Software, MA, USA). Representative immunoblots are shown in Figure S12A, and the corresponding normalized band intensities for all variants across proteinase K concentrations in Figure S12B. (B) Phosphatase activity of ACP3 proteins measured by using 4-methylumbelliferyl phosphate as a substrate. Enzymatic activity was assessed in culture supernatants and corresponding solubilized cell fractions collected 24 h after transfection. Activities were measured using samples adjusted to equivalent ACP3 protein amounts based on quantitative immunoblot analysis. Endogenous phosphatase activity, determined from untransfected cells processed in parallel, accounted for ∼60% of the WT signal and was subtracted from all measurements to obtain ACP3-specific activity. Reactions were terminated by alkali quenching, and liberated 4-methylumbelliferone was quantified by fluorescence (excitation, 365 nm and emission, 445 nm). Data represent the mean ± SD of *n* = 3 independent experiments. N.A.; not analyzed due to absence of ACP3 in the supernatant. Statistical significance versus WT was assessed by Dunnett’s multiple-comparisons test (∗∗∗*p* < 0.001; n.s., not significant) using GraphPad Prism (ver. 9.5.1, GraphPad Software, MA, USA).

Collectively, these findings indicate that loss of *N*-glycosylation at Asn333 compromises the conformational stability of ACP3, whereas disruption at other glycosylation sites (e.g., Asn220) exerts more modest effects, supporting a site-specific and nonredundant role for the Asn333 glycan in maintaining protein folding and stability. The enhanced protease sensitivity of the N333Q and T335M proteins is consistent with the ER accumulation and aggregate formation observed by fluorescence and electron microscopy, and provides a structural basis for the functional impairment of these variants described further.

### Site-specific effects of *N*-glycosylation on ACP3 phosphatase activity

To evaluate the functional consequence of site-specific *N*-glycosylation, we first measured the phosphatase activity of the ACP3 variant proteins using 4MU-phosphate as a substrate. Prior to enzymatic measurements, culture supernatants and cell lysates were adjusted to equivalent ACP3 protein amounts based on quantitative immunoblot analysis; thus, the measured activities reflect differences in ACP3-specific catalytic activity rather than in protein abundance. Enzymatic activity was quantified by fluorescence detection of liberated 4-methylumbelliferone in both the culture supernatants and the corresponding cell lysates (Figure 6B). To accurately quantify ACP3-specific phosphatase activity, background values from non-transfected controls were subtracted from all measurements.

Both the culture supernatant and lysate from cells expressing WT ACP3 exhibited robust phosphatase activity. Samples from cells expressing the N94Q variant retained substantial phosphatase activity, although a modest reduction relative to WT ACP3 was observed. These findings suggest that the Asn94-linked glycan may make a minor contribution to optimal enzymatic function but is not essential for ACP3 catalytic competence. Cells expressing N220Q ACP3 also retained substantial enzymatic activity in the supernatant and lysate, despite the partially reduced secretion efficiency of this variant. These results suggest that the N220 glycan contributes to optimal folding and trafficking, but is not strictly required for intrinsic catalytic competence once the protein achieves its native conformation.

In striking contrast, cells expressing either the N333Q or T335M variant showed no detectable phosphatase activity in the intracellular fraction. Given that these proteins accumulate within the ER and display enhanced protease susceptibility, the absence of enzymatic activity is most consistent with severe conformational destabilization rather than a selective defect in the catalytic site itself.

Taken together, these data demonstrate that *N*-glycosylation exerts site-specific effects on ACP3 function: whereas the glycans at Asn94 and Asn220 modulate secretion efficiency to varying degrees, that at Asn333 is essential for productive folding and acquisition of enzymatic activity.

### The Asn333 *N*-glycan stabilizes ACP3 through extensive intramolecular interactions

Although the crystal structure of human ACP3 has been determined, electron density corresponding to the *N*-glycan moieties has been only partially resolved (Figure 7A). To elucidate the structural contribution of each *N*-glycan, we performed all-atom MD simulations of glycosylated ACP3 based on the crystal structure (PDB: 1CVI). Glycan structures were modeled based on the predominant glycan classes identified by glycomic and glycoproteomic analyses, together with previous reports^16^^,^^17^^,^^18^ (Figure 7B). Because ACP3 exhibits considerable site-specific glycan microheterogeneity, it was not practical to model all experimentally observed glycoforms. Consequently, the simulations were designed to evaluate the relative structural contributions of glycans at different sites rather than to reproduce the complete glycan microheterogeneity of ACP3. Representative glycans were selected to capture the predominant class at each site, while allowing direct comparison of site-specific structural effects. Asn333 was modeled with Man_6_GlcNAc_2_, corresponding to the predominant glycoform identified at this site.^17^ In contrast, Asn94 and Asn220 were modeled using representative biantennary complex-type glycans, reflecting the dominant complex-type nature of glycans at these sites. These structures were intentionally simplified and selected to provide comparable glycan size and branching, thereby minimizing confounding effects arising from differences in glycan length or composition. Sialic acid residues were not included because of their heterogeneous linkage patterns and variable occupancy.^17^

**Figure 7:**
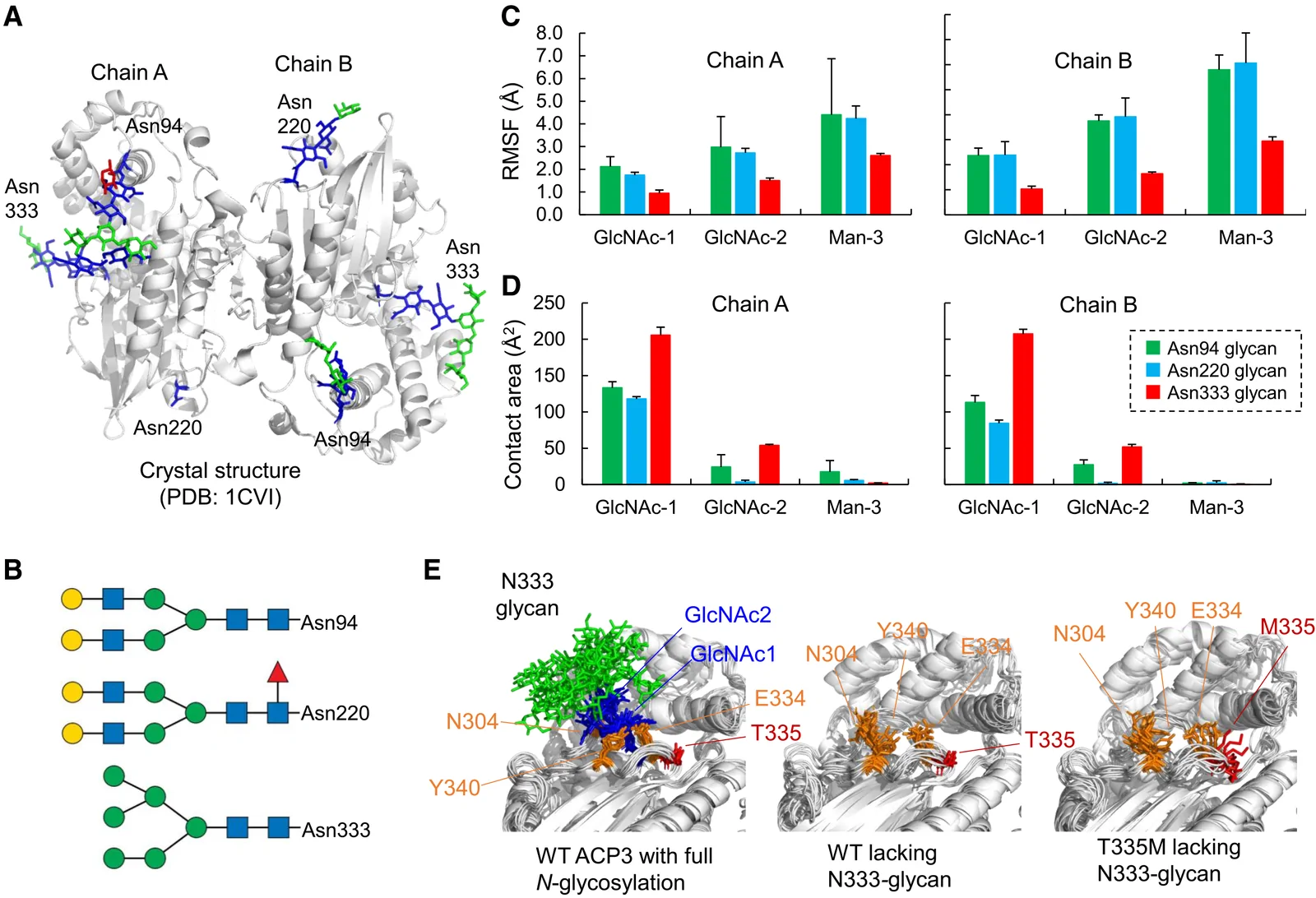
The Asn333-linked *N*-glycan stabilizes ACP3 through extensive intramolecular interactions (A) Crystal structure of human ACP3 (PDB: 1CVI) showing partially resolved electron density corresponding to *N*-glycan moieties. (B) The *N*-glycan structures modeled at Asn94, Asn220, and Asn333, which were used as the initial structure for MD simulations. Glycans are depicted using the Symbol Nomenclature for Glycans (SNFG) system.^27^d-GlcNAc, blue square; d-Man, green circle; d-Gal, yellow circle; and l-Fuc, red triangle. (C) Root-mean-square fluctuation (RMSF) (Å) of individual sugar residues derived from MD simulations. RMSF values, calculated after fitting trajectories to the protein backbone, are the mean ± SD for chain A and chain B from three independent simulations. The Asn94 glycan is shown in green, the Asn220 glycan in blue, and Asn333 glycan in red. (D) Glycan-protein contact areas calculated from differences in solvent-accessible surface area between the fully glycosylated protein and structures lacking individual glycan residues. Values are the mean ± SD for chain A and chain B from three independent simulations. The coloring scheme is the same as in (C). (E) Representative snapshots from MD simulations comparing fully *N*-glycosylated ACP3 (left), ACP3 lacking the Asn333 glycan (middle), and the T335M variant lacking Asn333 glycosylation (right). Structures are superimposed at 10-ns intervals over a 100-ns trajectory to illustrate conformational stability around Asn333 and neighboring residues. The core glycan residues (GlcNAc and Man) are shown in blue and green, respectively; surrounding amino acid residues (Asn304, Glu334, and Tyr340) are shown in brown.

Because ACP3 forms a homodimer in the crystal structure, all simulations were performed on the dimeric assembly. To ensure the robustness of the results, three independent MD simulations were conducted for each system. In each simulation, the root-mean-square deviations (RMSDs) of backbone residues largely stabilized at 30 ns, indicating that the system had reached equilibrium (Figures S13–S15). Structural parameters were analyzed separately for chain A and chain B in each trajectory, and comparable trends were observed across independent runs and between the two protomers.

To evaluate glycan flexibility, root-mean-square fluctuation (RMSF) values were calculated for individual sugar residues (Figure 7C). Consistently, the innermost GlcNAc residue of the Asn333 glycan exhibited markedly lower RMSF values (0.9 ± 0.1 Å in chain A and 1.0 ± 0.1 Å in chain B) as compared with that at Asn94 (2.1 ± 0.4 Å in chain A and 2.4 ± 0.3 Å in chain B) or Asn220 (1.7 ± 0.1 Å in chain A and 2.4 ± 0.5 Å in chain B). A similar trend was observed for subsequent core residues (GlcNAc-2 and Man-3), indicating that the Asn333 glycan core is conformationally restrained. This reduced mobility suggests that there are stable intramolecular interactions between the Asn333 glycan and the polypeptide backbone.

To quantify these interactions, glycan-protein contact areas were calculated from differences in the solvent-accessible surface area between glycosylated ACP3 and the protein without each sugar residue (Figure 7D). Among all glycosylation sites within the ACP3 dimer, the GlcNAc-1 residue at Asn333 exhibited the largest contact area with the polypeptide (210 ± 16 Å^2^ in chain A and 209 ± 14 Å^2^ in chain B), substantially exceeding those of Asn94 (138 ± 10 Å^2^ in chain A and 104 ± 10 Å^2^ in chain B) and Asn220 (114 ± 7 Å^2^ in chain A and 92 ± 12 Å^2^ in chain B). These results indicate that the Asn333 glycan forms extensive surface contacts with the protein.

Structural inspection revealed that the Asn333 glycan core is nestled within a local network involving residues Asn304, Glu334, and Tyr340 (Figure 7E). Hydrogen bond analysis confirmed stable glycan-protein hydrogen bonding at GlcNAc-1 onto Asn333 (frequency >70%), whereas hydrogen bonds involving glycans at Asn94 or Asn220 were less persistent (Table 1). No stable inter-glycan hydrogen bonds were detected. Notably, MD simulations performed in the absence of the Asn333 glycan revealed increased fluctuations of Asn333 and its neighboring residues, including Asn304, Glu334, and Tyr340 (Figures 7E and S16–S20), indicating local destabilization of the surrounding structural elements.

**Table 1:** List of glycan-polypeptide hydrogen bonds observed during MD simulations of ACP3

Chain^a^	Glycosylation site	Sugar residue	Sugar atom	Amino acid residue	Residue atom	Frequency (%)
A	Asn220	GlcNAc1	N	Val218	O	54 ± 14
A	Asn333	GlcNAc1	O6	Asn304	Nδ	87 ± 2
A	Asn333	GlcNAc1	N	Glu334	Oε	78 ± 6
B	Asn94	GlcNAc1	N	Glu95	Oε	24 ± 12
B	Asn220	GlcNAc1	N	Val218	O	22 ± 18
B	Asn333	GlcNAc1	O6	Asn304	Nδ	77 ± 12
B	Asn333	GlcNAc1	N	Glu334	Oε	82 ± 1

a Only hydrogen bonds with an occurrence frequency of more than 20% are shown. Frequencies are reported as the mean ± standard deviation from three independent MD simulations.

Taken together, these data support a model in which the Asn333-linked high-mannose glycan acts as a “structural clamp”, stabilizing a specific surface region of ACP3 through extensive intramolecular contacts and hydrogen bonding. This structural stabilization is consistent with our experimental findings: loss of the Asn333 glycan resulted in ER retention, aggregation, increased protease susceptibility, and complete loss of enzymatic activity. In contrast, the comparatively smaller contact areas and higher flexibility of glycans at Asn220 and Asn94 suggest that these residues have more limited contributions to global structural stability. Consistent with this interpretation, disruption of the Asn94 or Asn220 glycosylation site did not alter the apparent dimeric state of secreted ACP3, indicating that these glycans are dispensable for oligomerization. Their primary contribution therefore seems to be toward optimization of protein maturation and secretion rather than to stabilization of the dimeric assembly. The site-specific glycan characteristics are summarized in Table 2.

**Table 2:** Structural and functional characteristics of site-specific *N*-glycans in human ACP3

Feature	Asn94-linked glycan	Asn220-linked glycan	Asn333-linked glycan
Main *N*-glycan type	complex-type	complex-type	high mannose-type
Contribution to protein secretion	dispensable	contributory	essential
Contribution to phosphatase activity	dispensable	largely dispensable	indirectly essential (via protein folding)
Conformational flexibility	high	high	low

### Clinical manifestations in patients harboring the ACP3 T335M variant

Our survey of in-house whole-exome sequencing variant data identified three unrelated individuals with undiagnosed disease carrying the p.T335M variant in the *ACP3* gene. The p.T335M variant was *de novo* and none of the healthy parents carried it. The allele frequency of the p.T335M *ACP3* gene in the gnomAD v4.1.0 database^28^ was 6.6 × 10^−4^, consistent with a rare variant.

Although the clinical findings for the identified individuals were heterogeneous, developmental delay and epilepsy were shared features among all three (Table 3). Case 1, a male child, presented with developmental delay, hypotonia, delayed myelination, intractable epilepsy, and cryptorchidism. Brain magnetic resonance imaging (MRI) revealed mild ventricular enlargement and a computed tomography (CT) scan showed dilated cardiomyopathy in a preschool child. Case 2, a male child, presented with severe intellectual disability, developmental delay, epilepsy, growth hormone deficiency, short stature, and characteristic facies. Brain MRI showed mild enlargement of the right ventricle in infancy. In addition to the *ACP3* gene, this patient was found to have a previously reported *de novo* pathogenic variant of the *YWHAG* gene, which is associated with developmental and epileptic encephalopathy 56 (OMIM: 617665) and specific learning disability.^29^^,^^30^ Case 3, a female infant, presented with severe, psychomotor developmental delay, electroencephalographic abnormalities, epilepsy, and paralysis of the extraocular and facial muscles. Brain CT scan revealed calcifications in the midbrain, pons, and medulla oblongata.

**Table 3:** Clinical features of three unrelated patients carrying the *de novo* ACP3 p.T335M variant

Case	Sex	Variant status	Core neurological features	Neuro-imaging findings	Additional clinical features	Additional genetic findings
1	male	*de novo*, hetero-zygous	developmental delay, hypotonia, intractable epilepsy, and delayed myelination	mild ventricular enlargement (MRI)	dilated cardiomyopathy and cryptorchidism	none reported
2	male	*de novo*, hetero-zygous	severe intellectual disability, developmental delay, and epilepsy	mild right ventricular enlargement (MRI)	growth hormone deficiency, short stature, and characteristic facies	*de novo* pathogenic variant in *YWHAG*
3	female	*de novo*, hetero-zygous	severe psychomotor developmental delay, epilepsy, EEG abnormalities, extraocular, and facial muscle paralysis	brainstem calcifications (midbrain, pons, and medulla)	–	none reported

Epilepsy and developmental delay/mental retardation (underlined) were observed in all three individuals.

EEG, electroencephalography; MRI, magnetic resonance imaging.

The molecular analyses presented here demonstrated that the T335M substitution abolishes *N*-glycosylation at Asn333, leading to ER retention, intraluminal aggregation, conformational destabilization, and complete loss of phosphatase activity. Given that ACP3 functions as a homodimer, incorporation of a misfolded subunit may interfere with dimer assembly or structural stability, potentially amplifying the functional deficit in heterozygous individuals. Whether the Asn333-linked *N*-glycan contributes directly to ACP3 homodimer assembly remains to be determined. Given the extensive structural stabilization provided by this glycan, as revealed by our MD simulations, disruption of dimer formation or dimer stability represents a plausible additional mechanism underlying the functional defects of the N333Q and T335M variants.

Beyond its established expression in the prostate, *ACP3* is expressed in multiple tissues, including the brain, kidney, liver, lung, skeletal muscle, placenta, salivary gland, spleen, thyroid, and thymus.^11^^,^^12^^,^^31^^,^^32^ The protein is also expressed in the central nervous system, where its phosphatase activity may play a functional role in neural physiology.^11^^,^^12^ Behavioral abnormalities and enlargement of the lateral ventricles have been observed in *ACP3* knockout mice.^12^ Notably, ventricular enlargement was also observed in Cases 1 and 2, raising the possibility that ACP3 dysfunction contributes to this phenotype.

In sensory neurons in the dorsal root ganglion, ACP3 functions as an ecto-nucleotidase, acting in parallel with NT5E to convert extracellular AMP to adenosine.^33^^,^^34^^,^^35^ Adenosine acts as a neuromodulator that suppresses neuronal excitability^36^ and has been shown to inhibit vascular endothelial calcification.^37^^,^^38^ Indeed, NT5E deficiency has been associated with arterial and joint calcification.^37^^,^^39^ Although adenosine production in the brain is supported by multiple enzymes in addition to ACP3, the calcification observed in case 3 raises the possibility that impaired ACP3 function may contribute to this phenotype.

Collectively, these findings suggest that ACP3 folding defects and loss of activity may disrupt critical phosphorylation regulatory pathways within neurons (Figures 8A and 8B). However, the precise mechanistic relationship between epilepsy and ACP3 dysfunction, observed in all cases, requires further investigation.

**Figure 8:**
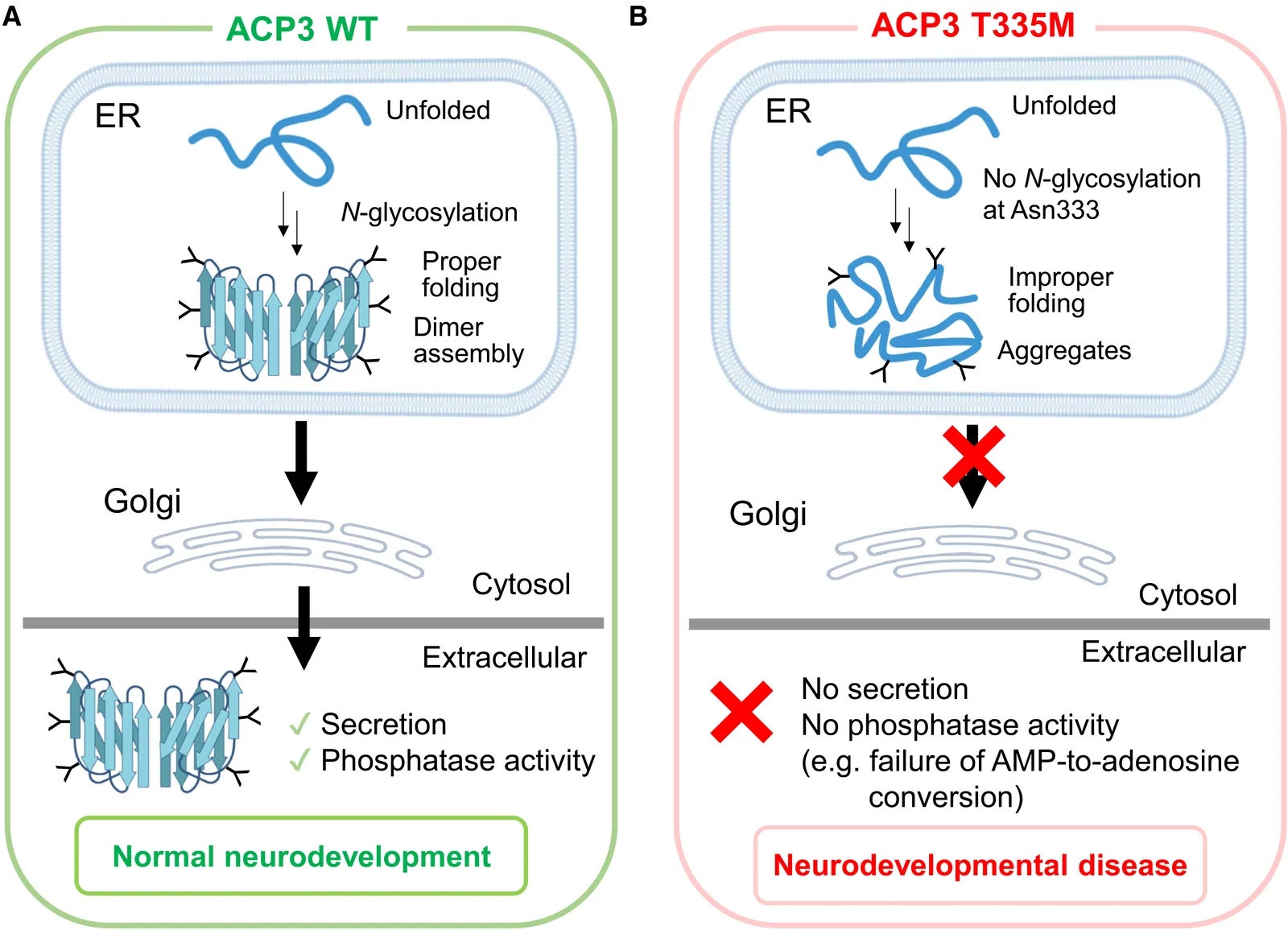
Proposed mechanism linking Asn333 *N*-glycan deficiency to ACP3 dysfunction and neurological phenotypes (A) In WT ACP3, *N*-glycosylation at Asn94, Asn220, and Asn333 promotes proper folding and structural stability, facilitating efficient secretion and normal phosphatase activity in the extracellular space. (B) In the T335M variant, *N*-glycosylation at Asn333 is abolished, resulting in structural destabilization, intracellular retention, and loss of enzymatic activity. Impaired secretion and/or intracellular aggregation of ACP3 may disrupt cellular homeostasis and contribute to the neurological manifestations observed in affected individuals.

### Limitations of the study

This study has several limitations. First, our biochemical and structural analyses were performed using recombinant ACP3 expressed in mammalian cells (Expi293F/HEK293). Although the site-specific glycosylation pattern of recombinant ACP3 broadly recapitulated that reported for endogenous ACP3, this heterologous expression system may not fully reflect the glycan processing, folding kinetics, or protein quality-control environment in tissues that naturally express ACP3, such as the prostate and central nervous system. Second, patient-derived ACP3 protein was unavailable from the three individuals carrying the T335M variant, which precluded direct biochemical characterization of the variant protein *in vivo*. Instead, the structural and functional consequences of T335M were evaluated using the recombinant expression system. Finally, although the three unrelated individuals carrying the T335M variant showed overlapping clinical features, including epilepsy and developmental delay, the limited number of individuals prohibited definitive genotype-phenotype correlations. Further studies using additional affected individuals and patient-derived biological materials will be required to establish a mechanistic link between ACP3 dysfunction and the observed neurodevelopmental phenotypes.

In summary, this study has identified Asn333-linked *N*-glycosylation as a chemically encoded structural determinant of ACP3 stability and enzymatic competence. This glycan-mediated stabilization is disrupted in the T335M variant of ACP3, providing a molecular framework that connects defective protein folding to neurodevelopmental abnormalities. More broadly, our findings suggest that individual *N*-glycans can function as chemically encoded structural stabilizers that operate hierarchically rather than redundantly within a single glycoprotein. In this context, site-specific *N*-glycan heterogeneity encodes different structural information, with distinct glycans exerting unequal contributions to conformational stability and proteostasis. This principle extends beyond ACP3 and may represent a general mechanism by which glycan diversity fine-tunes protein folding efficiency and functional robustness in mammalian systems.

## Resource availability

### Lead contact

Requests for further information, resources, and reagents should be directed to and will be fulfilled by the lead contact, Yoshiki Yamaguchi (yyoshiki@tohoku-mpu.ac.jp).

### Materials availability

Plasmids generated in this study are available from the lead contact upon request, subject to a completed materials transfer agreement.

### Data and code availability

• Mass spectrometry glycoproteomics and glycomics data have not been deposited in a public repository but are available from the lead contact upon request.
• This paper does not report original code.
• Any additional information required to reanalyze the data reported in this paper is available from the lead contact upon request.

## Acknowledgments

This work was supported by the 10.13039/100009619Japan Agency for Medical Research and Development (10.13039/100009619AMED) under grant nos. JP22ek0109446, JP25ek0109815, and JP26ek0109886. The molecular dynamics simulations were carried out using the supercomputing facilities of the Cyberscience Center, Tohoku University. We thank Dr. Suyong Re (National Institute of Biomedical Innovation, Health and Nutrition) for valuable assistance with the MD simulations. We are grateful to Sachie Matsubara and Junri Hayakawa (Laboratory for Electron Microscopy, Kyorin University School of Medicine) for their assistance with electron microscopy. GaLSIC is one of the Joint Usage/Research Centers supported by the 10.13039/501100009056J-GlycoNet cooperative network, which is accredited by the 10.13039/501100001700MEXT. K.I., H.H., J.-i.F., K.A., A.T., K.A.-K., and S.N. are supported by the “Human Glycome Atlas Project” as one of the “Large-Scale Academic Frontier Promotion Projects” of 10.13039/501100001700MEXT. We would also like to thank NAI Inc. for their support with English language editing.

## Author contributions

Conceptualization, T.K., S.N., and Y.Y.; investigation, all authors; data curation, N.M., S.O., K.A.-K., and T.K.; writing – original draft, N.M., K.I., S.O., T.F., H.H., J.-i.F., Y.A., and Y.Y.; writing – review and editing: all authors; visualization, N.M., K.I., S.O., H.H., J.-i.F., and Y.A.; supervision, T.K., S.N., and Y.Y.; project administration, T.K., S.N., and Y.Y.; funding acquisition, T.K., S.N., and Y.Y.

## Declaration of interests

The authors declare no competing interests.

## STAR★Methods

### Key resources table

**Table:** 

REAGENT or RESOURCE	SOURCE	IDENTIFIER
**Antibodies**		

Anti 6×Histidine, Monoclonal Antibody(9C11), Peroxidase Conjugated	FUJIFILM Wako Pure Chemical Corp.	010–23181
Calreticulin antibody	GeneTex, Inc.	GTX00740
Peroxidase AffiniPure Goat Anti-Rabbit IgG (H + L)	Jackson ImmunoResearch Laboratories, Inc.	111–035-003; RRID: AB_2313567
Anti-His tag mouse monoclonal antibody	Proteintech Group Inc.	Cat# 66005-1-Ig; RRID: AB_11232599
Anti-calnexin (CANX) rabbit polyclonal antibody	GeneTex	Cat# GTX109669; RRID: AB_1949824
Anti-GRP94 (HSP90B1) rabbit polyclonal antibody	GeneTex	Cat# GTX103232; RRID: AB_1950516
Anti-GOLPH2 rabbit polyclonal antibody	GeneTex	Cat# GTX116154; RRID: AB_11171591
Alexa Fluor 488-conjugated anti-mouse IgG	Thermo Fisher Scientific	Cat# A-11001; RRID: AB_2534069
Alexa Fluor 647-conjugated anti-mouse IgG	Thermo Fisher Scientific	Cat# A-21240; RRID: AB_2535809
Cy3-conjugated anti-rabbit IgG	Thermo Fisher Scientific	Cat# A10520; RRID: AB_10563288
Alexa Fluor 555-conjugated anti-rabbit IgG	Thermo Fisher Scientific	Cat# A-21428; RRID: AB_2535849
Anti-GAPDH antibody, peroxidase conjugated	FUJIFILM Wako Pure Chemical Corp.	Cat# 015–25473; RRID: AB_2665526

**Chemicals, peptides, and recombinant proteins**		

Vector pcDNA3.4-TOPO	Thermo Fisher Scientific	A14697
pRL-TK plasmid	Promega Corp.	E2241
M-PER Mammalian Protein Extraction	Thermo Fisher Scientific	78501
Immobilon Forte Western HRP Substrate	Merck KGaA	WBLUF0100
c-PAGEL *Neo* CN-520L (5–20%)	ATTO Corp.	2331500
Immobilon-P Membrane	Merck KGaA	IPVH00010
Tween 20	Nacalai tesque, Inc.	35624–15
Skim milk	Nacalai tesque, Inc.	31149–75
Iodoacetamide	FUJIFILM Wako Pure Chemical Corp.	095–02151
Tris(2-carboxyethyl)phosphine Hydrochloride (TCEP)	Nacalai tesque, Inc.	07277–61
Sequencing Grade Modified Trypsin	Promega Corp.	V5111
Ni^2+^ Sepharose 6 Fast Flow	Cytiva	17531801
PNGase F	New England Biolabs, Inc.	P0704S
CBB Stain One Super	Nacalai tesque, Inc.	11642
PNGase F Prime	N-Zyme Scientifics, LLC	NZS1
BlotGlyco 100C	Sumitomo Bakelite Co., Ltd.	BS-45408
2,5-Dihydroxybenzoic acid	Sigma-Aldrich	149357
C18 reverse-phase column	Nikkyo Technos, Co., Ltd.	NTCC-360
Fetal bovine serum	BioWest	Cat# S1810-500
1×Penicillin-Streptomycin	FUJIFILM Wako Pure Chemical Corp.	Cat# 168–23191
Lipofectamine 2000 Transfection Reagent	Thermo Fisher Scientific	Cat# 11668500
Opti-MEM I Reduced Serum Medium	Thermo Fisher Scientific	Cat# 31985070
Cellstain Hoechst 33258 Solution	FUJIFILM Wako Pure Chemical Corp.	Cat# 343–07961
Proteinase K	Nacalai tesque, Inc.	29442–14
Quick Start Bradford 1× Dye Reagent	Bio-Rad Laboratories, Inc.	500–0205
Ammonium bicarbonate	Merck KGaA	09830-500G
Dithiothreitol	Nacalai tesque, Inc.	14112–52
4-Methylumbelliferyl Phosphate	Tokyo Chemical Industry Co., Ltd.	P1060
SignalStain Boost IHC Detection Reagent (HRP, Mouse)	Cell Signaling Technology, Inc.	8125
3,3′-Diaminobenzidine (DAB)	FUJIFILM Wako Pure Chemical Corp.	040–27001
Paraformaldehyde	Nacalai Tesque Inc.	09154–14
Glutaraldehyde	TAAB Laboratories Equipment Ltd.	G011/1
Osmium tetroxide	Nissin EM	300–1
Epon 812	Laboratories Equipment Ltd.	T031
Uranyl acetate	Merck	8473
Lead citrate	TAAB Laboratories Equipment Ltd.	U001
Lysozyme	Nacalai Tesque, Inc.	22584–61
Ovalbumin	GE Healthcare	28403841B
Conalbumin	GE Healthcare	28403841A
Endo H (Endo Hf)	New England Biolabs	P0703L
Stripping buffer	FUJIFILM Wako Pure Chemical Corp.	193–16375

**Critical commercial assays**		

Gxpress 293 Transfection & Medium Kit	GMEP Inc.	GX293-MAK-0010
Renilla-Glo Luciferase Assay System	Promega Corp.	E2710

**Deposited data**		

Raw and analyzed data	N/A	N/A

**Experimental models: Cell lines**		

HEK293 cells	JCRB Cell Bank	Cat# JCRB9068; RRID: CVCL_0045
Expi293F cells	Thermo Fisher Scientific	A14527; RRID:CVCL_D615

**Software and algorithms**		

ImageJ	Wayne Rasband (NIH)	https://imagej.net/ij/index.html
Imaris (ver.9.3.1)	Oxford Instruments	RRID: SCR_007370; http://www.bitplane.com/imaris/imaris
Image Lab software (ver.6.1)	Bio-Rad Laboratories, Inc.	https://www.bio-rad.com/
Discovery Studio 2021	Dassault Systèmes	https://www.3ds.com/
AlphaFold	AlphaFold Server	https://alphafoldserver.com/welcome
PyMOL Molecular Graphics System (ver. 2.5)	Schrödinger, LLC	https://www.pymol.org/
GENESIS 1.7.0	Kobayashi et al.^40^	https://mdgenesis.org/
FlexAnalysis software (ver. 3.4)	Bruker Daltonics	https://www.bruker.com/en.html
Proteome Discoverer (v3.0 SP1)	Thermo Fisher Scientific	https://www.thermofisher.com/jp/en/home.html
Byonic (v5.11.27)	Protein Metrics	https://www.proteinmetrics.com/
GraphPad Prism (ver. 9.5.1)	GraphPad Software	https://www.graphpad.com/
Microsoft Excel	Microsoft	https://www.microsoft.com/

**Other**		

ChemiDoc Touch MP imaging system	Bio-Rad	https://www.bio-rad.com/
NanoDrop One	Thermo Fisher Scientific	https://www.thermofisher.com/jp/en/home.html
UltrafleXtreme MALDI-TOF mass spectrometer	Bruker Daltonics	https://www.bruker.com/en.html
EASY-nLC 1200 system coupled to a Q Exactive mass spectrometer	Thermo Fisher Scientific	https://www.thermofisher.com/jp/en/home.html
LSM700 confocal laser microscope	Carl Zeiss	https://www.zeiss.com/corporate/en/home.html
Poly-l-lysine-coated 8-well chamber slides	Matsunami Glass	SCS-N18
JEM-1011 transmission electron microscope	JEOL Ltd.	https://www.jeol.co.jp/
SpectraMax iD microplate reader	Molecular Devices, LLC.	https://www.moleculardevices.com/
Alliance e2695 HPLC system	Waters	https://www.waters.com/
COSMOSIL 5Diol-120-II column (300 × 7.5 mm i.d.)	Nacalai Tesque, Inc.	38050–51
Micro Vac MV-100	TOMY	https://www.digital-biology.co.jp/manufactured/
miVac DUO	SP Genevac	https://scientificproducts.com/

### Experimental model and study participant details

#### Cell lines

HEK293 cells (JCRB Cell Bank, Cat# JCRB9068) and Expi293F cells (Thermo Fisher Scientific, Cat# A14527) are both human embryonic kidney-derived cell lines. Both cell lines were maintained as described in the method details section. HEK293 cells were obtained from Japanese Collection of Research Bioresources Cell Bank where the test for mycoplasma contamination was examined. Expi293F cells were purchased from Thermo Fisher Scientific and were certified mycoplasma-negative by the vendor at the time of purchase.

#### Human participants

Whole-exome sequencing data were obtained from approximately 3,000 patients with rare and undiagnosed diseases and their parents (trio sequencing), as previously described.^21^ This study identified three unrelated individuals harboring *de novo* heterozygous ACP3 p.T335M variants: two male children (Cases 1 and 2) and one female infant (Case 3); detailed clinical features, including age at assessment and developmental stage, are provided in Table 3. The study was approved by the Ethics Committee of the National Center for Child Health and Development (Approval No. 2020-326), and written informed consent was obtained from the parents or legal guardians of all participants. Given the small number of affected individuals identified (*n* = 3; two male, one female), the influence of sex on phenotypic severity or penetrance could not be statistically assessed.

### Method details

#### Survey of ACP3 variants among patients with rare and undiagnosed disease

We searched for individuals with variants in the *N*-linked glycosylation site of ACP3 based on in-house whole-exome sequencing variant data from 3000 patients with rare and undiagnosed diseases and their parents, as previously described.^41^

#### Design and construction of expression plasmids

The amino acid sequence of human prostatic acid phosphatase (ACP3; UniProt: P15309-1), corresponding to the canonical secreted isoform, was used as the reference sequence. A DNA fragment encoding residues 33–386, representing the signal peptide-cleaved soluble catalytic domain of human ACP3, was cloned into mammalian expression vector pcDNA3.4-TOPO (Thermo Fisher Scientific) with an N-terminal interleukin-2 (IL-2) signal peptide (MYRMQLLSCIALSLALVTNS) to direct secretion and a C-terminal octa-histidine (His_8_) tag to facilitate purification.

Site-directed variants were designed to disrupt individual *N*-glycosylation sites by substituting Asn94, Asn220, or Asn333 with glutamine (N94Q, N220Q, N333Q), or by introducing the disease-associated T335M substitution, which abolishes the canonical Asn333 glycosylation sequon. In addition, a double mutant (N94Q/N220Q) was generated to enable site-specific characterization of the *N*-glycan attached to Asn333.

All constructs were codon-optimized for mammalian expression, chemically synthesized (Thermo Fisher Scientific), and expressed in mammalian cells to preserve native-like *N*-glycan processing and site-specific glycosylation patterns.

#### Protein expression and purification

Expi293F cells were maintained in 30 mL of HE400AZ medium (GMEP Inc.) at 37 °C under 8% CO_2_ with orbital shaking at 125 rpm. Transient transfection was performed at a density of 3.0×10^6^ cells/mL using 27 μg of pcDNA3.4 plasmid encoding ACP3 together with 3 μg of pRL-TK plasmid (Promega) encoding *Renilla* luciferase as a transfection efficiency control. Transfections were carried out using 293 Complexing Solution, Gxpress 293 Tf reagents, and Gxpress 293 Enhancer (GMEP Inc.) according to the manufacturer’s instructions.

Transfection efficiency was assessed 48 h after transfection by measuring *Renilla* luciferase activity. Cells were lysed in M-PER™ Mammalian Protein Extraction Reagent (Thermo Fisher Scientific) and incubated with an equal volume of Renilla-Glo™ Luciferase Assay Reagent (Promega Corp.) for 10 min, before chemiluminescence was measured. Luminescence signals were used to adjust sample loading volumes for SDS-PAGE, thereby normalizing ACP3 expression levels across samples before immunoblot analysis.

Recombinant ACP3 expression was evaluated in both culture supernatants and cell lysates by SDS-PAGE and western blotting using a peroxidase-conjugated anti-6×His monoclonal antibody (clone 9C11; FUJIFILM Wako). Signals were visualized with Immobilon Forte Western HRP Substrate (Millipore) and quantified using ImageJ.^42^

For protein purification, culture supernatants were collected 7 days after transfection by centrifugation at 2,000×*g* for 10 min at 4 °C. Supernatants were dialyzed three times against 1 L of phosphate-buffered saline (PBS; 10 mM Na_2_HPO_4_, 1.8 mM KH_2_PO_4_, 137 mM NaCl, 2.7 mM KCl, pH 7.4), filtered and applied to a 1-mL Ni Sepharose 6 Fast Flow column (Cytiva) pre-equilibrated with PBS. After washing with 5 mL of PBS containing 50 mM imidazole, bound proteins were eluted stepwise with 2-mL fractions of PBS containing increasing imidazole concentrations (100, 200, 300, 400, and 500 mM). Eluted fractions were assessed by SDS-PAGE to confirm purity and integrity. The concentrations of purified ACP3 proteins were determined by absorbance at 280 nm (NanoDrop One^C^; Thermo Fisher Scientific) using a calculated molar extinction coefficient of 61,685 L mol^−1^ cm^−1^.

#### Western blotting

Proteins separated by SDS-PAGE were transferred onto a PVDF membrane (Millipore) in standard Tris-glycine transfer buffer (25 mM Tris-HCl, 192 mM glycine, and 20% (v/v) methanol) for 60 min at 12 V. Membranes were washed three times with PBS containing 0.1% (v/v) Tween 20 (PBS-T), blocked with 5% (w/v) skim milk in PBS-T for 30 min at room temperature (RT), washed three times with PBS-T, and then incubated for 16 h at 4 °C with a peroxidase-conjugated anti-6×His monoclonal antibody (1:7,000 dilution in PBS-T). After washing with PBS-T three times, immunoreactive bands were detected by adding 800 μL of Immobilon Forte HRP substrate (Millipore) followed by visualization with a ChemiDoc Touch MP imaging system (Bio-Rad).

For quantitative comparison of ACP3 levels in cell lysates and culture supernatants, intracellular ACP3 signals were normalized to the corresponding GAPDH signals to correct differences in cell number across samples. After ACP3 detection, the PVDF membranes were stripped using Western blot stripping buffer (FUJIFILM Wako Pure Chemical, Japan), washed with PBS containing 0.1% Tween 20, and re-probed with a peroxidase-conjugated anti-GAPDH antibody (FUJIFILM Wako Pure Chemical, Osaka, Japan). Band intensities were quantified using the normalized intracellular ACP3 signals together with the corresponding culture supernatant ACP3 signals to evaluate the relative distribution of ACP3 between the intracellular and secreted fractions.

To detect calreticulin, samples were analyzed by SDS-PAGE followed by immunoblotting with rabbit anti-calreticulin antibody (GTX00740, GeneTex) and goat anti-rabbit antibody with HRP conjugation.

#### Size exclusion chromatography

Size-exclusion chromatography was performed on a Waters Alliance e2695 HPLC system controlled by the Empower 3 chromatography data system (Waters, Milford, MA, USA) using a COSMOSIL 5Diol-120-II column (300 × 7.5 mm i.d.; Nacalai Tesque, Kyoto, Japan). The mobile phase consisted of PBS delivered at a flow rate of 1.0 mL/min. Elution was monitored by UV absorbance at 280 nm. The column was calibrated using lysozyme (14 kDa), ovalbumin (44 kDa), and conalbumin (75 kDa) as molecular weight standards.

#### PNGase F and Endo H treatment of cell lysates

For PNGase F and Endo H analysis, M-PER-solubilized cell lysates were prepared from cells harvested 2 days after transfection with expression plasmid in an independent experiment. The lysates were subjected to deglycosylation using PNGase F (New England Biolabs) or Endo Hf (New England Biolabs) according to the manufacturer’s protocol. Specifically, equal amounts of cell lysates were mixed with Glycoprotein Denaturing Buffer (New England Biolabs), heated at 98 °C for 10 min, and cooled to room temperature. Deglycosylation was then performed by addition of GlycoBuffer (New England Biolabs), NP-40, and PNGase F or endo H at 37 °C for 16 h. Control samples were processed in parallel under identical conditions except that enzyme was omitted. The reactions were terminated by adding SDS-PAGE sample buffer and heating at 95 °C for 5 min. Samples were analyzed by SDS-PAGE and immunoblotting using an HRP-conjugated anti-His tag antibody as described above.

#### Preparation of protein *N*-glycans for focused protein glycomics

Focused protein glycomic analysis was performed as previously reported,^43^ with minor modifications. For WT ACP3, the purified protein was directly subjected to reduction, alkylation, and tryptic digestion. For the N94Q/N220Q double mutant, low-level contaminants were detected by SDS-PAGE after affinity purification; therefore, the protein was further separated by SDS-PAGE using a precast 5%–20% polyacrylamide gel (c-PAGEL *Neo*, ATTO, Tokyo, Japan) at 21 mA. After Coomassie Brilliant Blue staining (CBB Stain One Super, Nacalai tesque, Kyoto, Japan), the band corresponding to the ACP3 N94Q/N220Q mutant was excised and subjected to in-gel reduction, alkylation, and tryptic digestion at 37 °C for 16 h.

For both proteins, tryptic peptides were treated with *N*-glycosidase F (PNGase F Prime™, N-Zyme Scientifics, Doylestown, PA, USA) at 37 °C for 16 h to release *N*-linked glycans for glycomic analysis.

#### Purification and derivatization of *N*-glycans by glycoblotting and aminolysis-SALSA

Chemoselective enrichment of *N*-glycans from WT ACP3 and the N94Q/N220Q mutant was performed using glycoblotting in combination with aminolysis-based sialic acid linkage-specific alkylamidation (SALSA), as previously described.^44^^,^^45^^,^^46^ Released *N*-glycans were captured on hydrazide-functionalized BlotGlyco beads via selective conjugation of their reducing ends, allowing removal of peptides and other contaminants.

After on-bead processing, *N*-glycans were released and derivatized with aminooxy-Trp-Arg through an imine exchange reaction to enhance detection sensitivity and ionization efficiency. Excess derivatization reagent was removed by hydrophobic interaction liquid chromatography elution plate before matrix-assisted laser desorption/ionization time-of-flight mass spectrometry (MALDI-TOF-MS) analysis. Glycan signals were quantified based on peak areas in MALDI-TOF-MS spectra.

#### MALDI-TOF MS analysis

Purified and derivatized *N*-glycans were mixed with 2,5-dihydroxybenzoic acid (10 mg/mL in 50% acetonitrile)^44^ as the matrix and subjected to analysis using an UltrafleXtreme MALDI-TOF mass spectrometer equipped with a reflector and operated with FlexControl software version 3.4 (Bruker Daltonics, Bremen, Germany). Mass spectra were acquired in the positive-ion reflectron mode with an acceleration voltage of 20 kV, a reflector voltage of 20.8 kV, and a pulsed-ion extraction time of 320 ns. Spectral annotation was performed using FlexAnalysis software version 3.4.

#### Trypsin digestion of ACP3 for LC-MS and LC-MS/MS analysis

Lyophilized WT ACP3 (40 μg), N94Q (15 μg), or N220Q (3 μg) was dissolved in 100 mM ammonium bicarbonate (34 μL) and subjected to reduction with dithiothreitol (final concentration 4 mM) at 60 °C for 30 min, followed by alkylation with iodoacetamide (11 mM) at RT for 30 min in the dark.

Proteins were digested with sequencing-grade modified trypsin (Promega, WI, USA) at an enzyme-to-substrate ratio of approximately 1:30 (w/w) and incubated at 37 °C for 3.5 h. The reaction was quenched by acidification with trifluoroacetic acid (final concentration 0.5% [v/v]). After centrifugation to remove insoluble material, the supernatant containing tryptic peptides was collected and directly subjected to liquid chromatography–MS (LC-MS) and LC-MS/MS analyses.

For the analysis of intracellular N333Q and T335M ACP3, M-PER-solubilized cell lysates were separated by SDS–PAGE, and ACP3 was subjected to in-gel trypsin digestion. After SDS–PAGE followed by Coomassie Brilliant Blue (CBB) staining, the ACP3 band (∼50 kDa) was excised, diced, and destained with 50% acetonitrile in 100 mM ammonium bicarbonate at 37 °C for 10 min. The gel pieces were dehydrated with acetonitrile, dried in a vacuum centrifuge (Micro Vac MV-100; TOMY, Tokyo), and incubated overnight (15–18 h) at 37 °C with sequencing-grade modified trypsin (10 ng/μL in 100 mM ammonium bicarbonate, pH 8.5). Peptides were subsequently extracted with 50% acetonitrile/0.1% trifluoroacetic acid, isopropanol/formic acid/acetonitrile/water (15:20:25:40, v/v/v/v), and 80% acetonitrile. The combined extracts were dried under vacuum and subjected to LC-MS and LC-MS/MS analyses.

#### LC-MS and LC-MS/MS analyses

Peptide samples were analyzed by using an EASY-nLC 1200 system coupled to a Q Exactive mass spectrometer equipped with a nanoelectrospray ionization source (Thermo Fisher Scientific), as described previously.^20^^,^^47^ Peptides were separated on a C18 reverse-phase column (75 μm × 125 mm, NTCC-360; Nikkyo Technos) using a linear gradient of solvent B (0.1% formic acid in 80% acetonitrile) against solvent A (0.1% formic acid in water) at a constant flow rate. The gradient was programmed as follows: 0–30 min, 0%–35% B; 30–35 min, 35%–95% B; and 35–45 min, 95% B.

The mass spectrometer was operated in positive ion data-dependent acquisition mode using a top 10 MS/MS method. Full MS (MS1) spectra were acquired at a resolution of 70,000 (*m/z* 380–4,000) with an automatic gain control (AGC) target of 3×10^6^. MS/MS spectra were generated by higher-energy C-trap dissociation^48^ at a resolution of 17,500 with an AGC target of 5×10^5^, isolation window of 1.6 *m/z*, maximum injection time of 150 ms, and normalized collision energy of 27. Dynamic exclusion was set to 15 s. For quantitative analysis, full scan LC-MS measurements were performed under identical chromatographic and instrumental conditions.

Raw data were processed using Proteome Discoverer (v3.0 SP1, Thermo Fisher Scientific). Database searches were conducted against the UniProt/Swiss-Prot human reference proteome (release 2024_03). Trypsin was specified as the proteolytic enzyme with allowance for up to two missed cleavages. Precursor and fragment mass tolerance were set to 6 ppm and 20 ppm, respectively. Carbamidomethylation of cysteine was defined as a fixed modification, while oxidation of methionine and phosphorylation of serine/threonine were considered variable modifications. Peptide-spectrum matches were filtered to a false discovery rate (FDR) of 1%.

Glycopeptide identification was performed using Byonic™ (v5.11.27, Protein Metrics)^49^ in stand-alone mode. Searches were conducted against the built-in *N*-glycan library. Only glycopeptides satisfying a 1% FDR threshold and a Byonic score higher than 300 were retained for further analysis. For glycopeptides identified from in-gel tryptic digests, a more permissive threshold (Byonic score >100) was applied owing to the limited amount of material recovered. Glycopeptide assignments were further validated by manual inspection of diagnostic oxonium ions in MS/MS spectra. Glycan structures were annotated according to the Symbol Nomenclature for Glycans (SNFG) system.^27^

#### Immunostaining of HEK293 cells expressing WT or mutant ACP3 protein

HEK293 cells (JCRB Cell Bank, Osaka, Japan) were cultured in DMEM (Thermo Fisher Scientific, Waltham, MA, USA) supplemented with 10% fetal bovine serum (BioWest, Nuaillé, France) and 1×Penicillin-Streptomycin (FUJIFILM Wako Pure Chemical Corporation, Osaka, Japan) at 37 °C in a humidified atmosphere with 5% CO_2_. Cells were seeded at a density of 4×10^6^ cells per 10-cm dish and incubated for 24 h. Expression vector (10 μg) encoding either WT or variant ACP3*,* and 20 μL of Lipofectamine® 2000 Transfection Reagent (Thermo Fisher Scientific), were each diluted separately in 1.5 mL of Opti-MEM I Reduced Serum Medium (Thermo Fisher Scientific), and then combined and incubated for 20 min at RT. The resulting transfection mixture (3 mL) was added to the culture medium and incubated for an additional 24 h.

The transfected cells were detached and reseeded on a poly-l-lysine-coated glass bottom dish (Matsunami Glass, Osaka, Japan) at densities of 1.2×10^5^ and 8.0×10^4^ cells, and incubated for 24 h. Cells were fixed with 4% paraformaldehyde in PBS for 10 min at RT, followed by three PBS washes. Cells were permeabilized in PBS containing 0.1% Triton X-100 and 1% bovine serum albumin (PBS-TB) for 30 min at RT, and incubated overnight at 4 °C with primary antibodies: anti-His tag mouse monoclonal antibody (Proteintech, Rosemont, IL, USA), anti-calnexin (CANX) rabbit polyclonal antibody (GeneTex, Irvine, CA, USA), anti-GRP94 (HSP90B1) rabbit polyclonal antibody (GeneTex), or anti-GOLPH2 rabbit polyclonal antibody (GeneTex). After three PBS washes, the cells were incubated for 1 h at RT with Hoechst 33258 (FUJIFILM Wako Pure Chemical Corporation) and Alexa Fluor 488-conjugated or Alexa Fluor 647-conjugated anti-mouse IgG, and Cy3-conjugated or Alexa Fluor 555-conjugated anti-rabbit IgG (Thermo Fisher Scientific). Confocal images were acquired using an LSM700 confocal laser microscope (Carl Zeiss, Oberkochen, Baden-Württemberg, Germany), and 3D reconstructions were performed using Imaris software (ver.9.3.1; Oxford Instruments, High Wycombe, UK). Statistical significance was evaluated by χ^2^ test (*n* = number of analyzed cells. ∗*p* < 0.0001).

#### Transmission electron microscopy

Transmission electron microscopy (TEM) was performed as previously described.^50^^,^^51^ HEK293 cells were seeded at densities of 2×10^5^ or 4×10^5^ cells in a 3.5-cm dish and incubated for 24 h prior to transfection as described above. Two days after transfection, cells were fixed with 2.5% glutaraldehyde (TAAB Laboratories Equipment, Aldermaston, UK) in 0.1 M phosphate buffer (pH 7.4) at RT and incubated overnight at 4 °C.

For immunoelectron microscopy, cells were seeded in 10-cm dishes at 4×10^6^ cells and transfected as described above. They were then reseeded on poly-l-lysine-coated 8-well chamber slides (Matsunami Glass) at 0.7×10^5^ or 0.9×10^5^ cells per well and incubated for 24 h. Cells were fixed in 4% paraformaldehyde (Nacalai Tesque, Kyoto, Japan) and 0.1% glutaraldehyde in PBS for 1 h at RT and then washed in PBS three times. After permeabilization in PBS-TB for 30 min at RT, the cells were incubated overnight at 4 °C with anti-His tag mouse monoclonal antibody (Proteintech). After three PBS washes, they were incubated with SignalStain® Boost IHC Detection Reagent (HRP, Mouse; Cell Signaling Technology, Danvers, MA, USA) for 30 min at RT with gentle agitation. After three PBS washes, the cells were incubated with 0.5 mg/mL of 3,3ʹ-diaminobenzidine (DAB; FUJIFILM Wako Pure Chemical Corporation) in PBS for 10 min at RT; 1% H_2_O_2_ (1/200 vol of DAB solution) was then added, followed by incubation for several minutes at RT. The cells were washed three times with PBS and post-fixed in 2.5% glutaraldehyde in PBS overnight at 4 °C.

The cells were then post-fixed with 1% osmium tetroxide (OsO_4_; Nissin EM, Tokyo, Japan) in 0.1 M phosphate buffer (pH 7.4) for 1 h at 4 °C, dehydrated in a graded series of ethanol, passed through propylene oxide, and embedded in Epon 812 (TAAB Laboratories Equipment). Ultrathin sections were stained with uranyl acetate (Merck, Darmstadt, Germany) and lead citrate (TAAB Laboratories Equipment), and examined using a JEM-1011 transmission electron microscope (JEOL, Tokyo, Japan).

#### Proteinase K resistance assay

A proteinase K resistance assay was used to assess the conformational stability and protease susceptibility of ACP3 variants. Expi293F cells transiently expressing each ACP3 construct were harvested and lysed in M-PER reagent (Thermo Fisher Scientific) according to the manufacturer’s protocol. After centrifugation to remove insoluble material, supernatants were collected for analysis. Total protein concentrations were determined by Bradford assay to ensure equal loading. Equal amounts of total protein (36 μg in 20 μL) were incubated with increasing concentrations of proteinase K (0, 5, 10, 20, and 40 ng/mL; Nacalai tesque, Kyoto, Japan) at 37°C for 1 h. Proteolysis was terminated by adding SDS sample buffer followed by heat denaturation. Samples were separated by SDS-PAGE and analyzed by western blotting using an anti-His tag antibody to detect ACP3. Band intensities were quantified by using Image Lab software (ver.6.1, Bio-Rad, CA, USA). Protease resistance was evaluated based on the relative preservation of full-length ACP3 across increasing proteinase K concentrations. Data represent the mean ± SD from four independent experiments. Statistical significance versus WT was assessed by Dunnett’s multiple comparisons test (∗*p* < 0.05, ∗∗*p* < 0.01) using GraphPad Prism (ver. 9.5.1, GraphPad Software, MA, USA).

#### Phosphatase activity assay

The phosphatase activity of the ACP3 variants was assessed by using 4-methylumbelliferyl phosphate (4MU-phosphate) as a fluorogenic substrate. To ensure accurate comparison among variants, ACP3 protein levels were first normalized based on quantitative western blot analysis. Culture supernatants or solubilized cell fractions were adjusted to equivalent ACP3 molar amounts by adding PBS or lysates derived from mock-transfected cells, respectively, thereby maintaining comparable background levels of endogenous phosphate activity across all conditions.

Reactions were performed in 96-well plates by incubating 10 μL of culture supernatant or solubilized cell fractions, 80 μL of 10 mM sodium phosphate buffer (pH 6.0), and 10 μL of 1 mM 4MU-phosphate (final volume 100 μL) at RT for 30 min. The reaction was terminated by adding 100 μL of 1 M Tris-HCl buffer (pH 10.0), which simultaneously enhanced the fluorescence of the released 4-methylumbelliferone. Fluorescence intensity was measured immediately using a SpectraMax iD microplate reader (Molecular Devices) with excitation at 365 nm and emission at 445 nm. Fluorescence measurements were performed within the linear detection range. Background signals from empty vector-transfected control cells were subtracted from all measurements. Statistical significance versus WT was assessed by Dunnett’s multiple comparisons test (∗∗∗*p* < 0.001) using GraphPad Prism (ver. 9.5.1, GraphPad Software, MA, USA).

#### Molecular dynamics simulations

All-atom MD simulations were performed to investigate the structural impact of site-specific *N*-glycosylation on human ACP3. The initial coordinates were derived from the X-ray crystal structure of human ACP3 (PDB: 1CVI, residues 33–374). Missing *N*-glycans were modeled using the CHARMM-GUI interface (https://www.charmm-gui.org/)^52^ as follows. At Asn333, the predominant high-mannose glycan identified by glycomic profiling was selected (Man_6_GlcNAc_2_). Because glycosylation at Asn94 and Asn220 is structurally heterogeneous, representative neutral complex-type glycans of comparable size to Man_6_GlcNAc_2_ were chosen based on previous experimental reports.^16^^,^^17^^,^^18^ This strategy facilitated structural comparison while minimizing size- and charge-related biases.

Simulations were performed using the CHARMM36 force field for proteins and glycans.^53^ The glycoprotein was placed in a cubic periodic box (119 Å × 119 Å × 119 Å) maintaining a minimum distance of 12 Å between protein images. The system was solvated with 48,538 explicit TIP3P water molecules^54^ and neutralized with counterions.

Energy minimization was carried out for 10,000 steps using the steepest descent algorithm. The system was gradually heated to 300 K under an *nVT* ensemble, followed by equilibration and production simulations under an *nPT* ensemble at 1 atm and 300 K. Production runs were performed for 100 ns with a 2-fs integration time step, employing periodic boundary conditions and particle mesh Ewald electrostatics.

Trajectory analyses were conducted using GENESIS 1.7.0 (RIKEN CCS, Kobe, Japan)^40^^,^^55^ and Visual Molecular Dynamics (VMD, version 1.9.4a51).^56^ Root-mean-square deviation (RMSD) was calculated relative to the initial structure. Root-mean-square fluctuation (RMSF) was obtained after least-squares fitting to the protein backbone. Hydrogen bonds were defined using geometric criteria: (1) donor and acceptor oxygens within 3.4 Å; (2) donor hydrogen–donor oxygen–acceptor oxygen angle within 30°; and (3) donor oxygen–donor hydrogen–acceptor oxygen angle greater than 120°. Solvent-accessible surface area was calculated using a 1.4 Å sphere probe and averaged over the 0–100 ns simulations.

Three independent simulations (runs #1–#3, *n* = 3) were performed for fully glycosylated ACP3 (Asn94, Asn220, and Asn333). Additional simulations were conducted for ACP3 lacking Asn333 glycosylation (*n* = 3) and for the T335M variant without Asn333-glycosylation (*n* = 3) to dissect the structural contribution of the Asn333 glycan and the impact of the disease-associated mutation.

### Quantification and statistical analysis

Statistical analyses were performed using GraphPad Prism (version 9.5.1) and Microsoft Excel. For comparisons of multiple ACP3 variants against WT (Figures 2B, 6A, and 6B), one-way ANOVA followed by Dunnett’s multiple comparisons test was used, with significance denoted as ∗*p* < 0.05, ∗∗*p* < 0.01, and ∗∗∗*p* < 0.001. For the categorical analysis of ACP3 subcellular localization (Figure 4B), a χ^2^ test was performed using Microsoft Excel, with significance denoted as ∗*p* < 0.0001. Statistical details for each experiment, including the exact value of n and the statistical test used, are provided in the corresponding figure legends. Data are presented as mean ± SD unless otherwise stated.
