# Supplementary material for: Loss of the Asn333 high-mannose N-glycan impairs ACP3 folding and drives disease-associated dysfunction
Source: iScience. 2026 Oct 7;29(10):117746. doi: 10.1016/j.isci.2026.117746 (PMC13676724; doi:10.1016/j.isci.2026.117746)
Supplement: Supplementary file 1 [file mmc1.pdf]

iScience, Volume *29*

Supplemental information

Loss of the Asn333 high-mannose *N*-glycan impairs ACP3 folding and drives disease-associated dysfunction

Noriyoshi Manabe, Kazuyoshi Itoh, Shiho Ohno, Ayumi Miura, Aika Miyata, Sora Yashima, Sachiko Komatsu, Tsutomu Fujimura, Hisatoshi Hanamatsu, Jun-ichi Furukawa, Kiyohiko Angata, Akira Togayachi, Kiyoko F. Aoki-Kinoshita, Yoshihiro Akimoto, Tadashi Kaname, Shoko Nishihara, and Yoshiki Yamaguchi

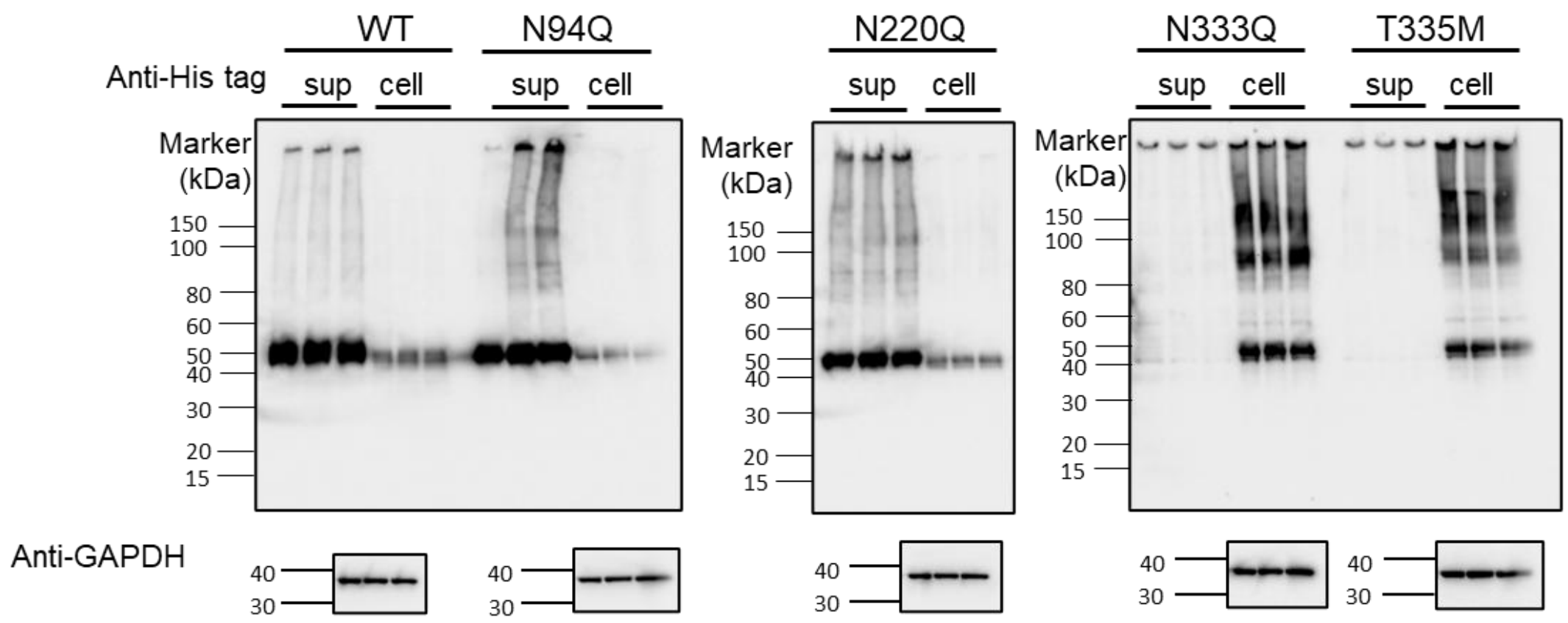

Supplementary Figure 1. Immunoblot analysis of ACP3 variants in culture supernatants and corresponding cell lysates collected 4 days after transfection. WT, N94Q, and N220Q proteins were predominantly detected in the supernatant; N333Q and T335M were largely retained in cells. GAPDH was analyzed as an internal control for the cell lysates to correct for differences in cell number among samples. Transfection efficiency was monitored by measuring *Renilla* luciferase activity from a co-transfected pRL-TK reporter plasmid. Quantitative analysis of the intracellular and secreted ACP3 levels, normalized using GAPDH and *Renilla* luciferase, is summarized in Figure 2B. Exposure times were 10 s for GAPDH; 6 s for WT, N94Q, and N220Q ACP3; and 120 s for N333Q and T335M ACP3.

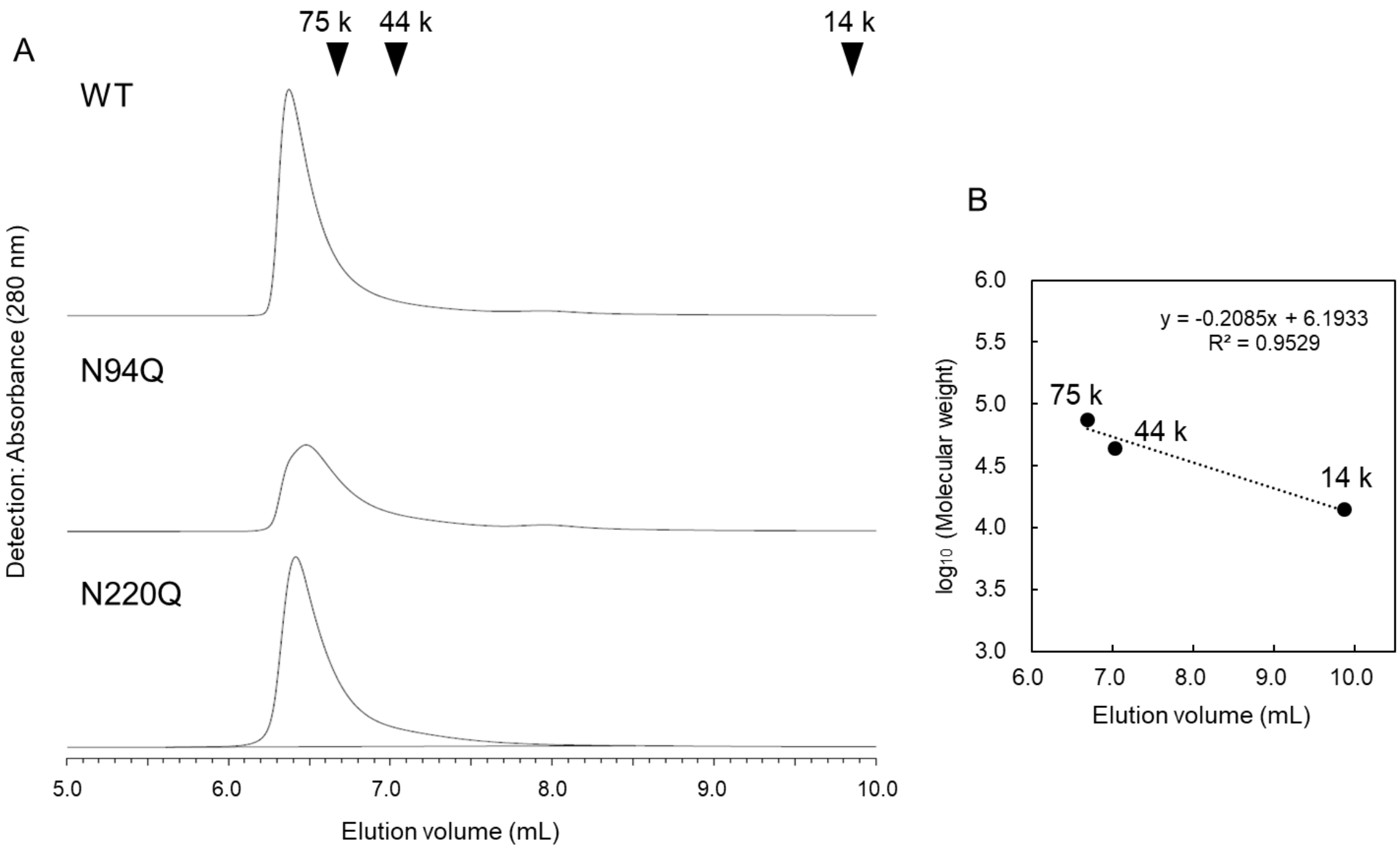

Supplementary Figure 2. Size-exclusion chromatography (SEC) analysis of purified WT, N94Q, and N220Q ACP3. (A) Representative SEC elution profiles. Purified WT, N94Q, and N220Q ACP3 were all eluted as a single major peak in a similar elution volume. (B) Calibration curve generated using molecular mass standards (lysozyme, 14 kDa; ovalbumin, 44 kDa; and conalbumin, 75 kDa). The apparent molecular masses of WT, N94Q, and N220Q ACP3 were estimated from the calibration curve and were consistent with a dimeric state.

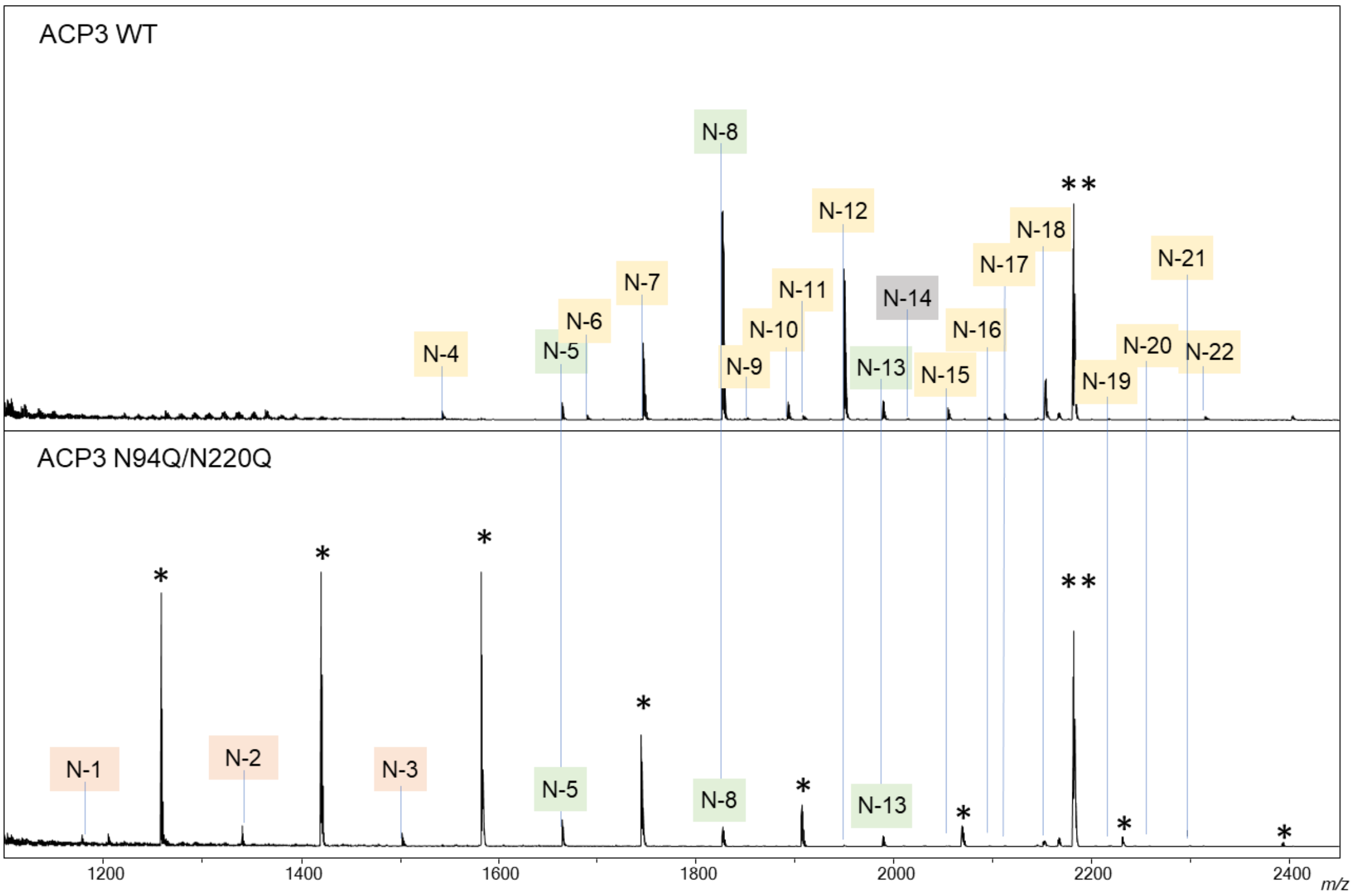

Supplementary Figure 3. MALDI-MS analysis of *N*-glycans released from ACP3. MALDI-MS spectra of *N*-glycans enzymatically released from WT ACP3 (upper panel) and the ACP3 N94Q/N220Q mutant (lower panel). Peaks marked with an asterisk (*) correspond to a hexose ladder, probably originating from cellular contaminants. Peaks marked with double asterisks (**) represent signals attributed to a non-glycan species.

No	m/z	Putative structure	Class	Wild type	Mutant
				Relative %	
N-1	1178.49	(Hex)2(HexNAc)2	PM	0.0	5.7
N-2	1340.55	(Hex)3(HexNAc)2	PM	0.0	12.6
N-3	1502.60	(Hex)1 + core	PM	0.0	9.8
N-5	1664.65	(Hex)2 + core	HM	2.6	22.8
N-8	1826.71	(Hex)3 + core	HM	34.0	19.7
N-13	1988.76	(Hex)4 + core	HM	3.8	13.2
N-14	2013.79	(Hex)2(HexNAc)1(Fuc)1 + core	Hyb	0.4	0.0
N-4	1543.63	(HexNAc)1 + core	Com	1.1	0.0
N-6	1689.68	(HexNAc)1(Fuc)1 + core	Com	0.8	0.0
N-7	1746.71	(HexNAc)2 + core	Com	10.9	0.0
N-9	1851.74	(Hex)1(HexNAc)1(Fuc)1 + core	Com	0.3	0.0
N-10	1892.76	(HexNAc)2(Fuc)1 + core	Com	3.0	0.0
N-11	1908.76	(Hex)1(HexNAc)2 + core	Com	0.8	0.0
N-12	1949.79	(HexNAc)3 + core	Com	26.6	2.0
N-15	2054.82	(Hex)1(HexNAc)2(Fuc)1 + core	Com	2.6	2.2
N-16	2095.84	(HexNAc)3(Fuc)1 + core	Com	0.6	2.0
N-17	2111.84	(Hex)1(HexNAc)3 + core	Com	1.5	2.3
N-18	2152.87	(HexNAc)4 + core	Com	8.6	0.0
N-19	2216.87	(Hex)2(HexNAc)2(Fuc)1 + core	Com	0.5	2.5
N-20	2257.90	(Hex)1(HexNAc)3(Fuc)1 + core	Com	0.5	1.9
N-21	2298.92	(HexNAc)4(Fuc)1 + core	Com	0.2	1.9
N-22	2314.92	(Hex)1(HexNAc)4 + core	Com	1.1	0.0
			Total	100.0	100.0

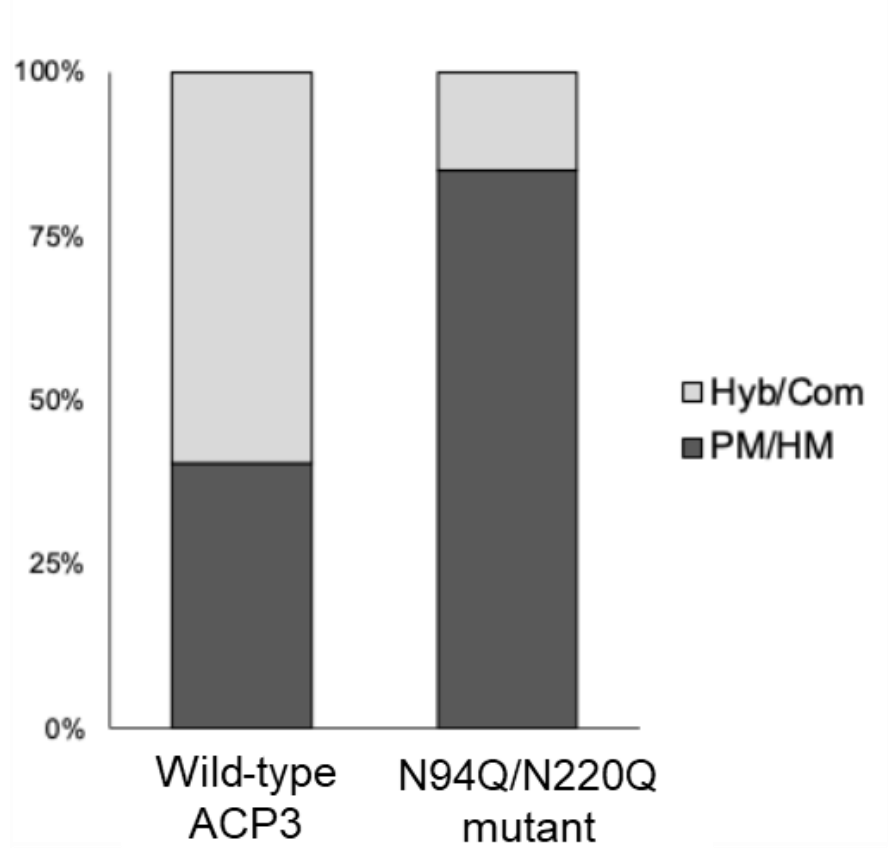

Supplementary Figure 4. Comparative *N*-glycan composition of WT ACP3 and the N94Q/N220Q mutant. Left, putative *N*-glycan structures. Right, relative abundance of hybrid/complex-type (Hyb/Com) and paucimannose/high-mannose-type (PM/HM) *N*-glycans in WT ACP3 and the N94Q/N220Q mutant.

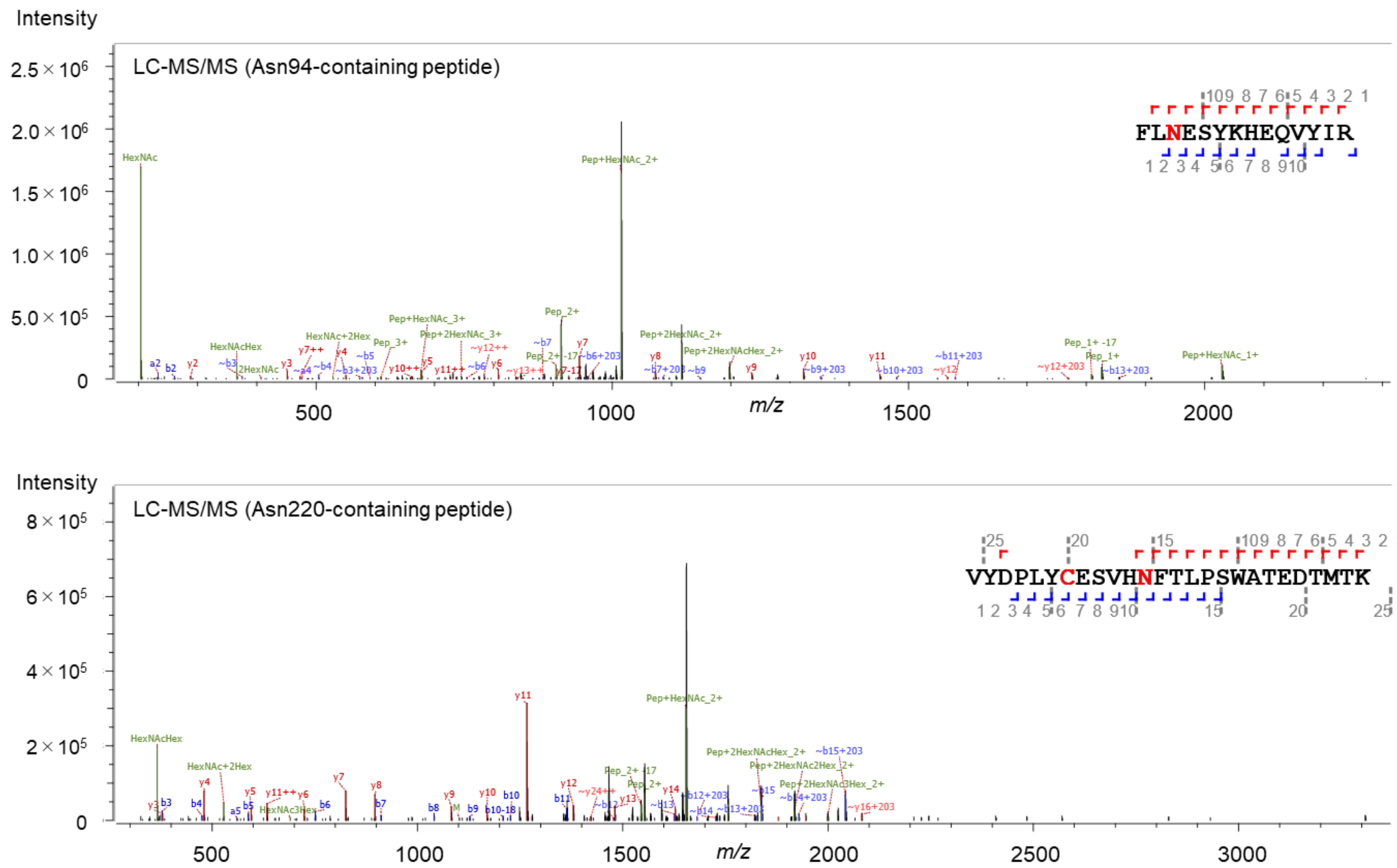

Supplementary Figure 5. Site-specific *N*-glycan profiling of Asn94 and Asn220 in WT ACP3. Representative LC-MS/MS spectra of tryptic glycopeptide containing Asn94 (top) and Asn220 (bottom) from WT ACP3. Diagnostic fragment ions and glycan compositions were used to assign site-specific *N*-glycan structures. A representative LC-MS/MS spectrum of tryptic glycopeptide containing Asn333 is shown in Figure 3.

Composition	class	relative %
Asn333		
HexNAc(2)Hex(6)	HM	78.2%
HexNAc(2)Hex(7)	HM	16.3%
HexNAc(2)Hex(8)	HM	2.5%
HexNAc(2)Hex(5)	HM	2.5%
Asn220		
HexNAc(4)Hex(3)	Hyb/Com	23.3%
HexNAc(5)Hex(3)	Hyb/Com	20.4%
HexNAc(6)Hex(3)	Hyb/Com	19.3%
HexNAc(3)Hex(3)	Hyb/Com	8.5%
HexNAc(5)Hex(4)	Hyb/Com	6.4%
HexNAc(2)Hex(5)	HM	5.2%
HexNAc(6)Hex(4)	Hyb/Com	4.5%
HexNAc(1)	Other	2.3%
HexNAc(6)Hex(6)	Hyb/Com	2.2%
HexNAc(4)Hex(3)Fuc(1)	Hyb/Com	1.9%
HexNAc(7)Hex(3)	Hyb/Com	1.8%
HexNAc(5)Hex(3)Fuc(1)	Hyb/Com	1.7%
HexNAc(6)Hex(3)Fuc(1)	Hyb/Com	1.4%
Asn94		
none	Unglycosylated	12.0%
HexNAc(5)Hex(3)	Hyb/Com	33.0%
HexNAc(4)Hex(3)	Hyb/Com	22.8%
HexNAc(6)Hex(3)	Hyb/Com	10.9%
HexNAc(2)Hex(5)	HM	6.1%
HexNAc(3)Hex(3)	Hyb/Com	5.8%
HexNAc(5)Hex(4)	Hyb/Com	2.9%
HexNAc(6)Hex(4)	Hyb/Com	2.1%
HexNAc(4)Hex(4)	Hyb/Com	1.1%

Supplementary Figure 6. Site-specific *N*-glycan composition of WT ACP3. The composition and relative abundance of glycans at each *N*-glycosylation site (Asn94, Asn220 and Asn333) are shown. Glycans with a low abundance (<1%) are omitted in the list.

ACP3 N94Q

Composition	class	relative %
Asn333		
HexNAc(2)Hex(6)	HM	72.8%
HexNAc(2)Hex(5)	HM	12.5%
HexNAc(2)Hex(4)	HM	8.5%
HexNAc(2)Hex(7)	HM	6.2%
Asn220		
HexNAc(5)Hex(3)	Hyb/Com	27.9%
HexNAc(6)Hex(3)	Hyb/Com	19.9%
HexNAc(4)Hex(3)	Hyb/Com	11.6%
HexNAc(5)Hex(3)Fuc(1)	Hyb/Com	9.2%
HexNAc(6)Hex(4)	Hyb/Com	8.2%
HexNAc(6)Hex(3)Fuc(1)	Hyb/Com	7.9%
HexNAc(4)Hex(3)Fuc(1)	Hyb/Com	6.6%
HexNAc(3)Hex(3)	Hyb/Com	3.8%
HexNAc(7)Hex(3)	Hyb/Com	2.7%
HexNAc(2)	Other	2.2%

Supplementary Figure 7. Site-specific *N*-glycan composition of ACP3 N94Q. The composition and relative abundance of glycans at each *N*-glycosylation site (Asn220 and Asn333) are shown. Glycans with a low abundance (<1%) are omitted in the list.

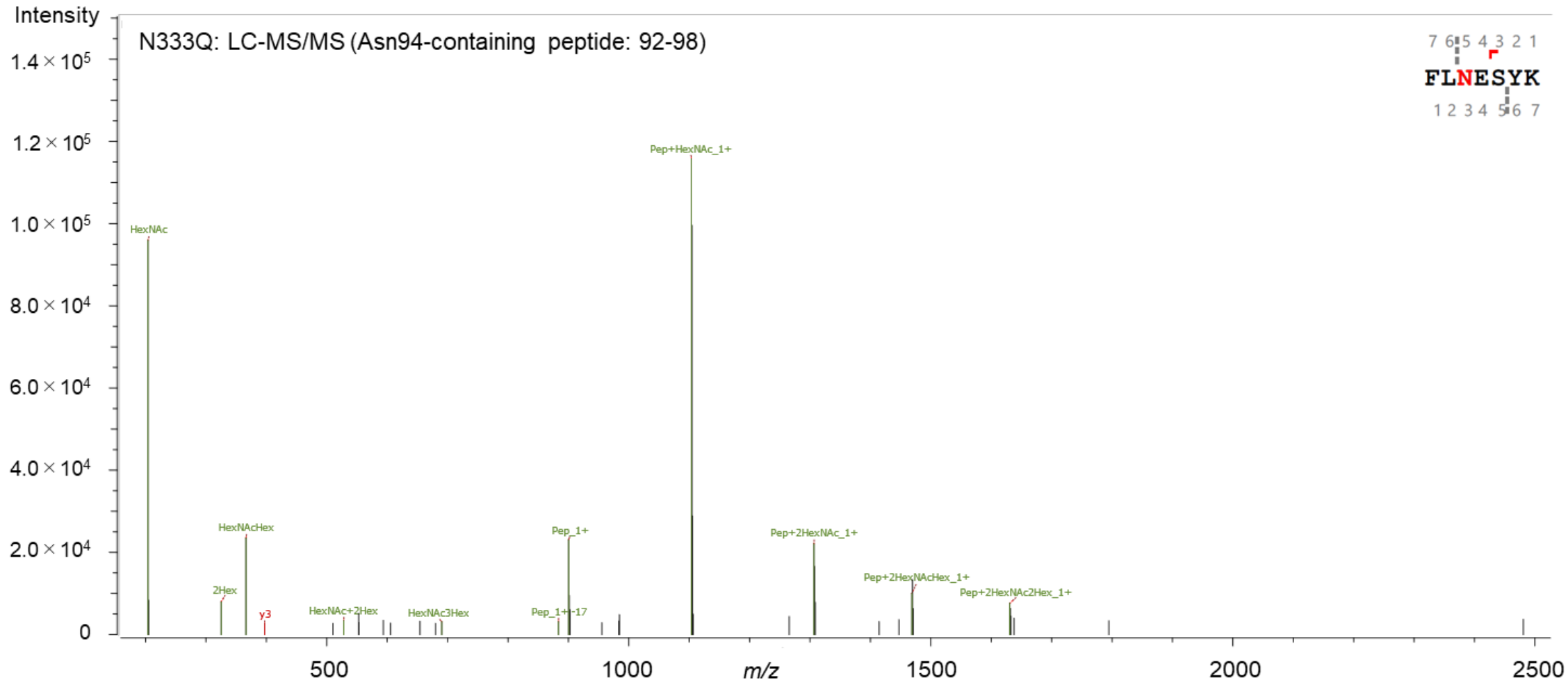

Supplementary Figure 8. Site-specific *N*-glycan profiling of Asn94 in ACP3 N333Q. Representative LC-MS/MS spectrum of a tryptic glycopeptide containing Asn94 from ACP3 N333Q. Diagnostic fragment ions and glycan compositions were used to assign site-specific *N*-glycan structure.

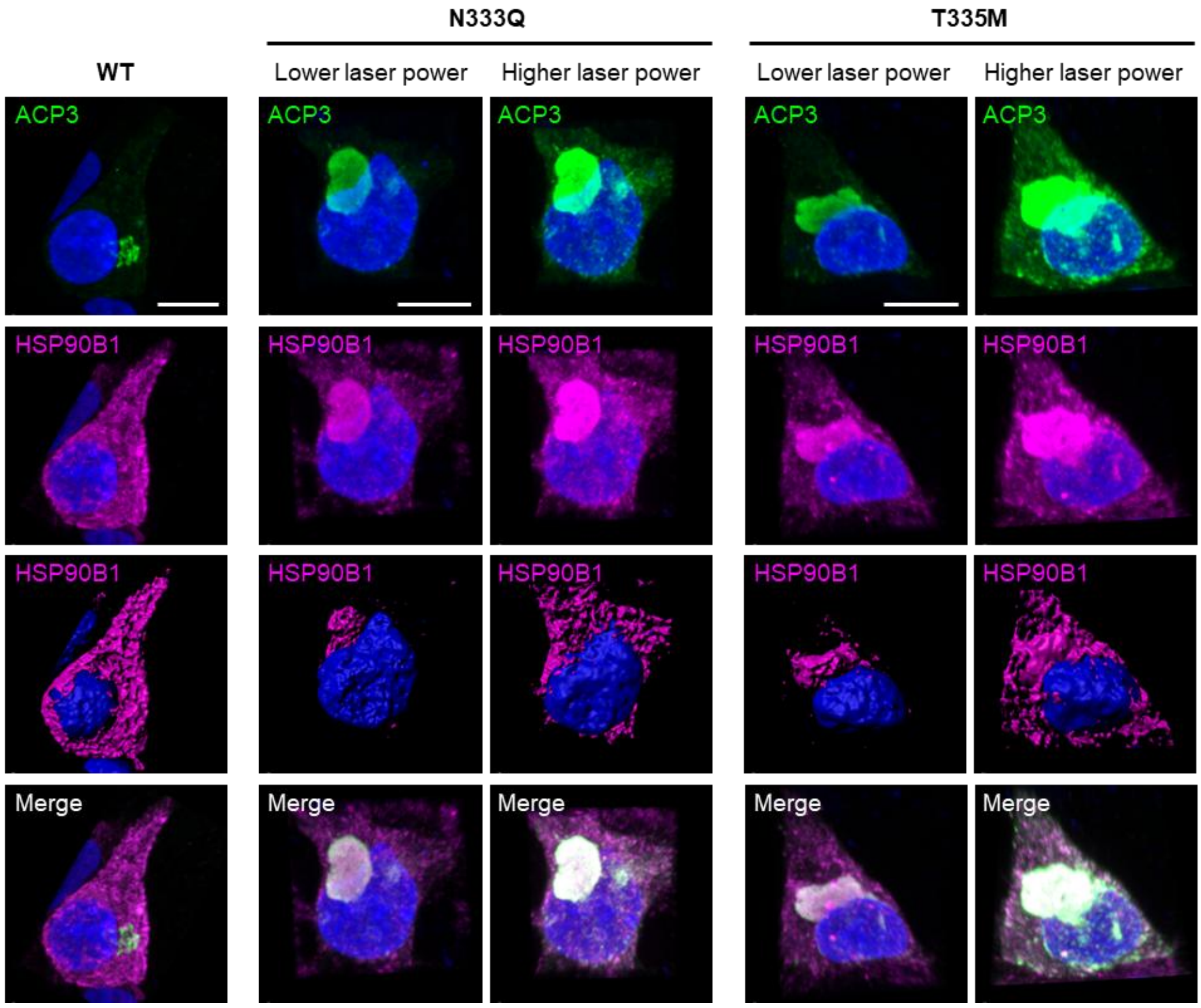

Supplementary Figure 9. Endoplasmic reticulum architecture in ACP3 aggregate-containing cells. Shown are 3D confocal images of HEK293 cells expressing WT or variant ACP3 proteins. Surface-rendered reconstructions are presented in the third row. ACP3 and nuclei were detected with anti-His tag antibody (green) and Hoechst 33258 (blue), respectively. The ER was labeled with anti-HSP90B1 antibody (magenta). Scale bars, 10 µm.

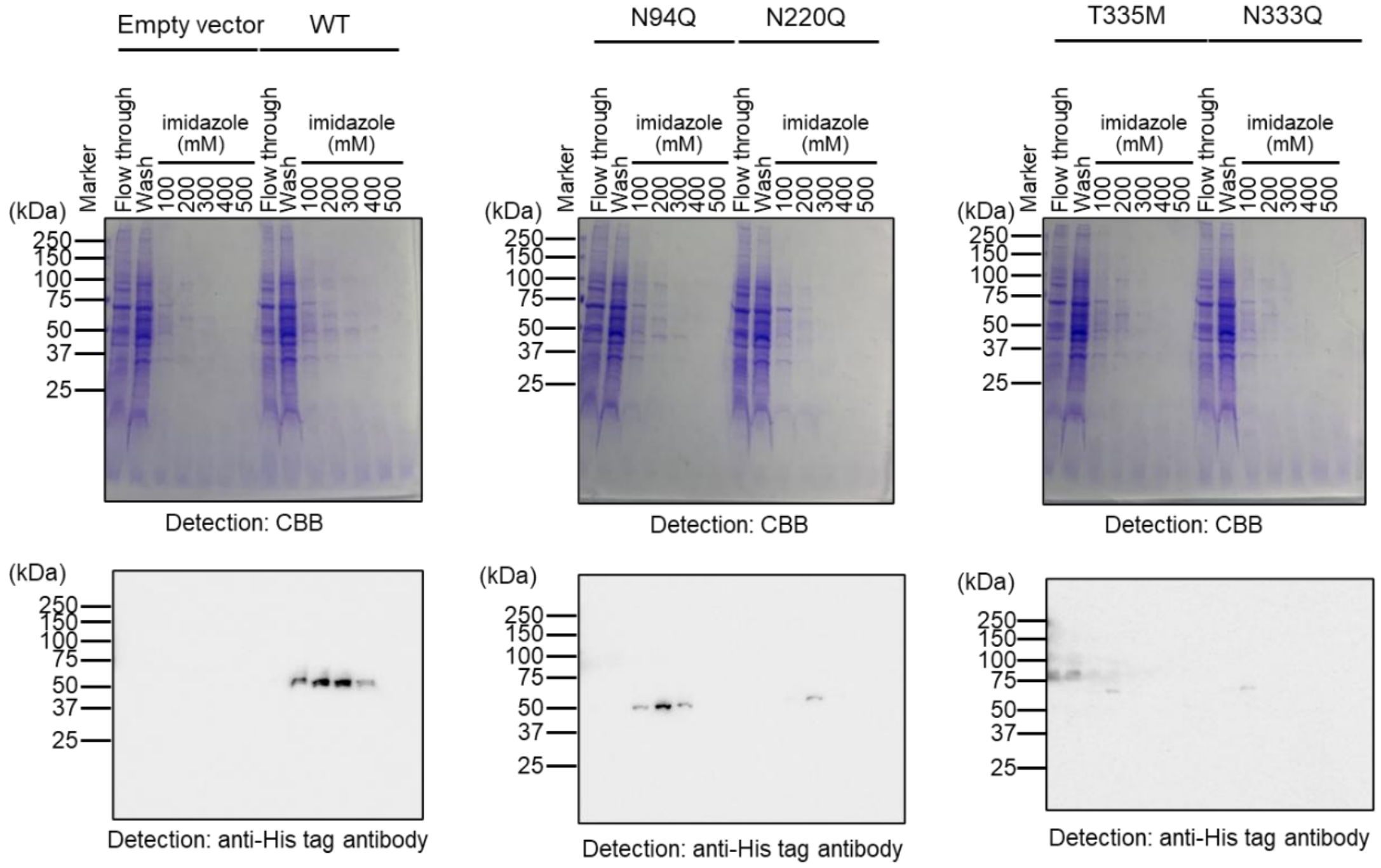

Supplementary Figure 10. Attempts to purify intracellular ACP3 from cell lysates. (A) Ni^{2+}-affinity purification of His_8-tagged ACP3 proteins from cell lysates. Expi293F cells were transfected with empty vector (control) or expression constructs encoding WT, N94Q, N220Q, N333Q, or T335M ACP3. Fractions eluted in increasing concentrations of imidazole (100–500 mM) were analyzed by SDS-PAGE. (B) Immunoblot analysis of ACP3 variants during Ni^{2+}-affinity purification. Proteins were separated by SDS-PAGE and detected using an HRP-conjugated anti-His tag antibody.

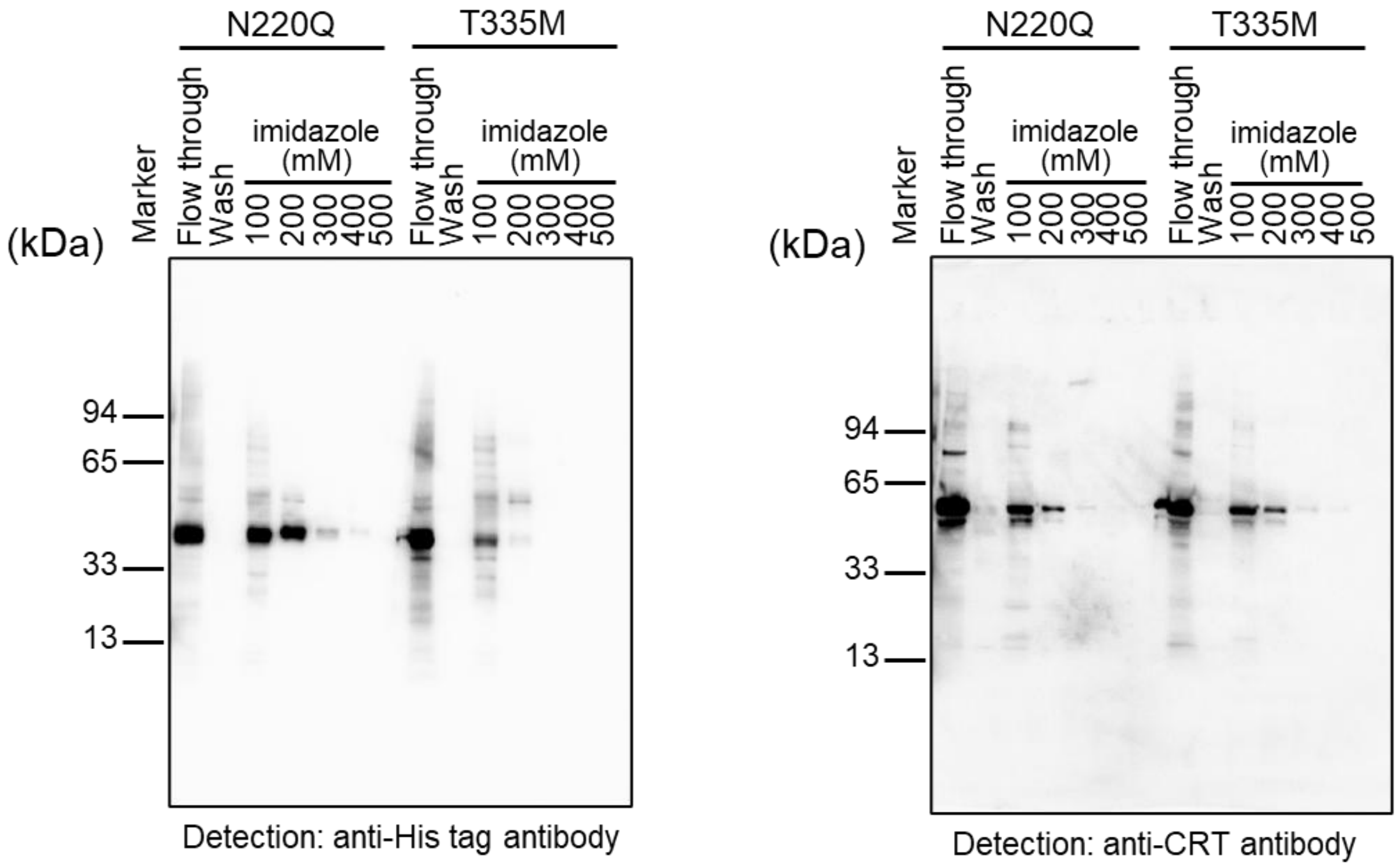

Supplementary Figure 11. Co-purification of calreticulin with glycan-deficient ACP3 variants. Cell lysates expressing N220Q or T335M ACP3 were purified by a Ni^{2+}-affinity column 2 days after transfection. Eluted fractions were analyzed by SDS-PAGE followed by immunoblotting with anti-His tag and anti-calreticulin (CRT) antibodies. Glycan-deficient N220Q and T335M variants exhibited reduced recovery and co-elution with CRT, suggesting association with ER-quality-control components.

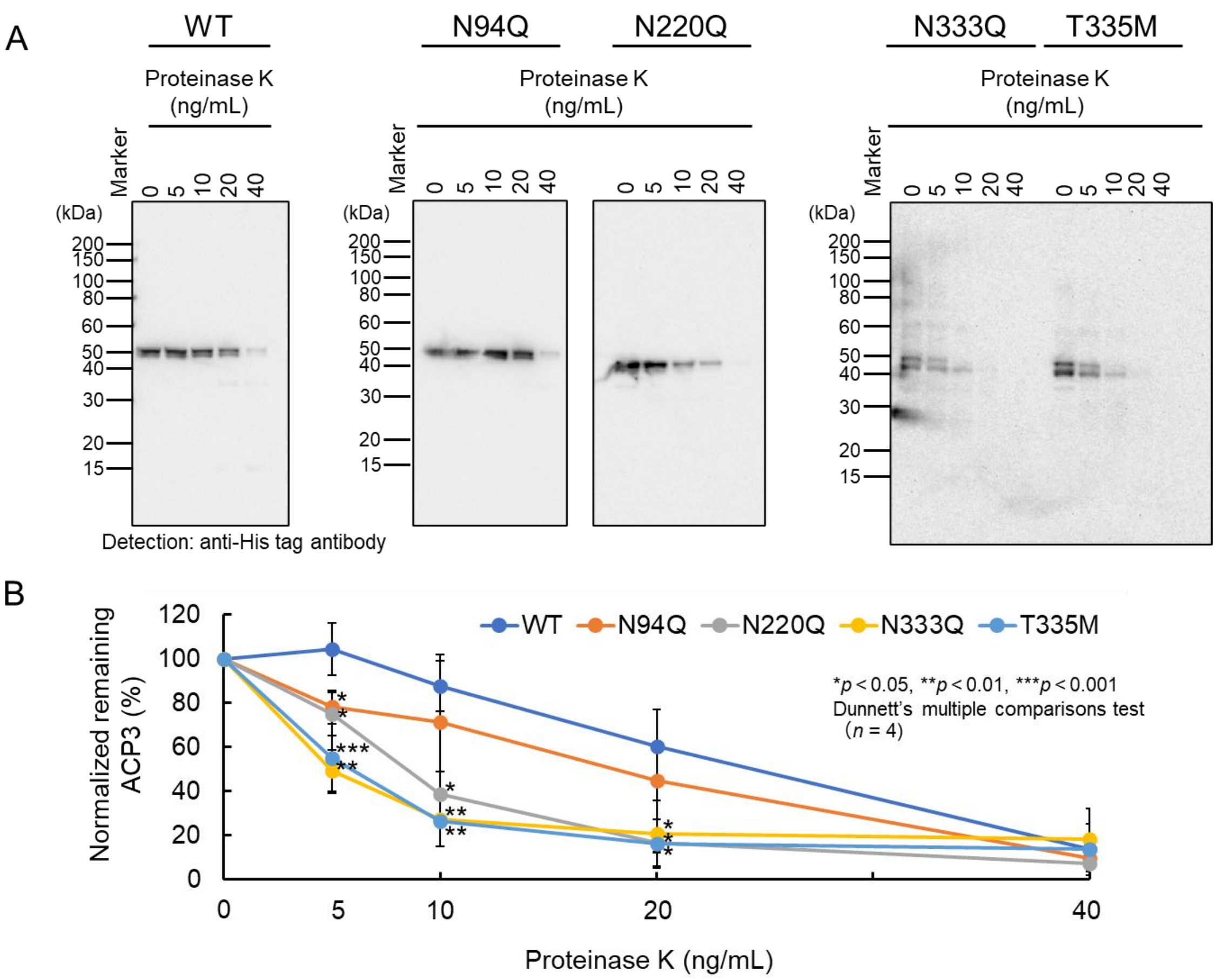

Supplementary Figure 12. Proteinase K sensitivity assay of ACP3 variants. WT ACP3 and the N94Q, N220Q, N333Q, and T335M variants were incubated with increasing concentrations of proteinase K for 1 h. Samples were analyzed by SDS-PAGE followed by immunoblotting using anti-His tag antibody (A). Band intensities were normalized to the untreated control and quantified from four independent experiments; mean ± SD values are plotted as a function of proteinase K concentration (B). Statistical significance versus WT was assessed by Dunnett's multiple comparisons test (*$p < 0.05$, **$p < 0.01$, ***$p < 0.001$) using GraphPad Prism (ver. 9.5.1, GraphPad Software, MA, USA).

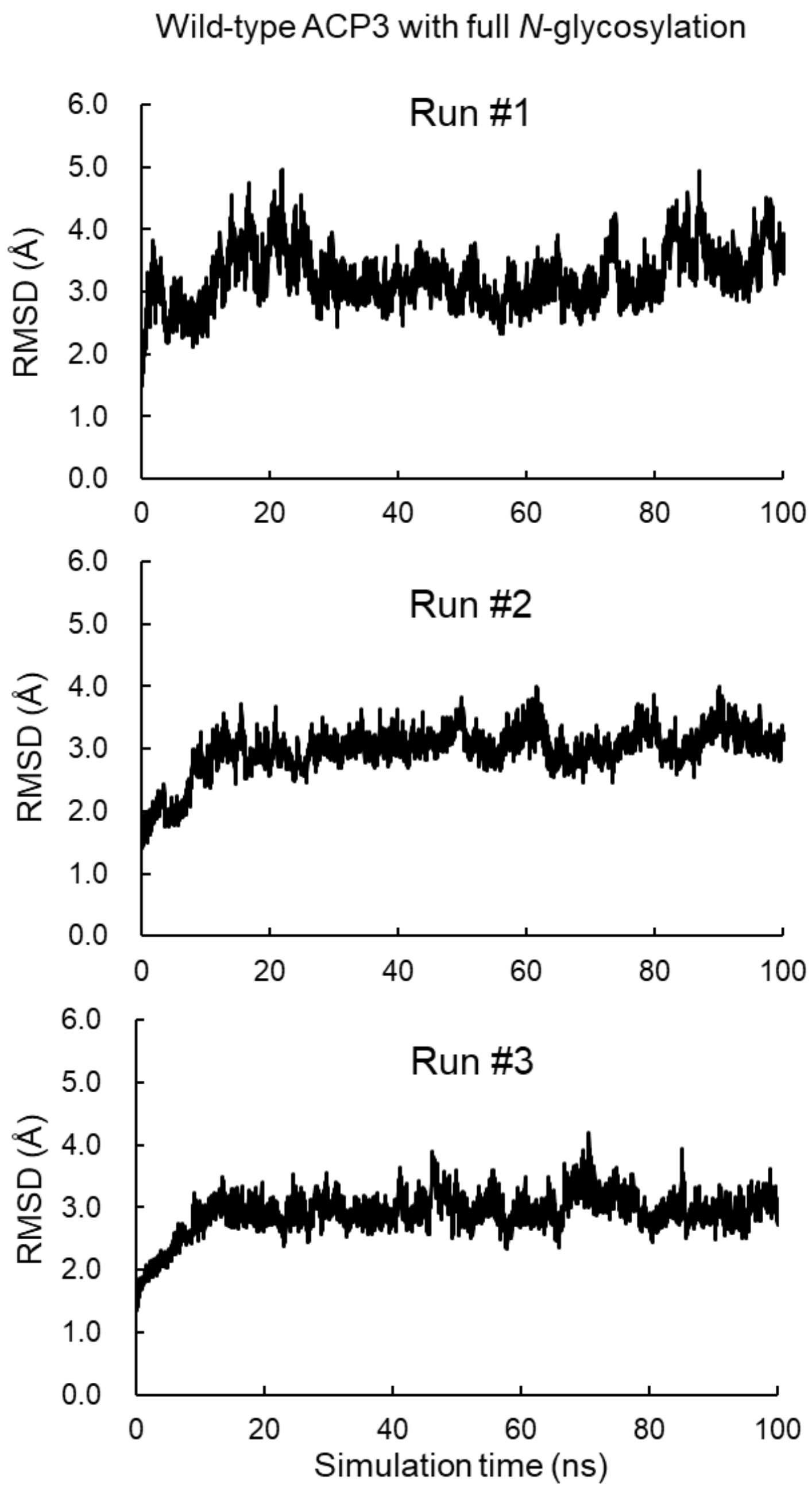

Supplementary Figure 13. Root-mean-square deviation (RMSD) analysis of fully glycosylated WT ACP3 during MD simulations. RMSD profiles are shown for WT ACP3 carrying three *N*-glycans at Asn94, Asn220, and Asn333 (n = 3 independent simulations).

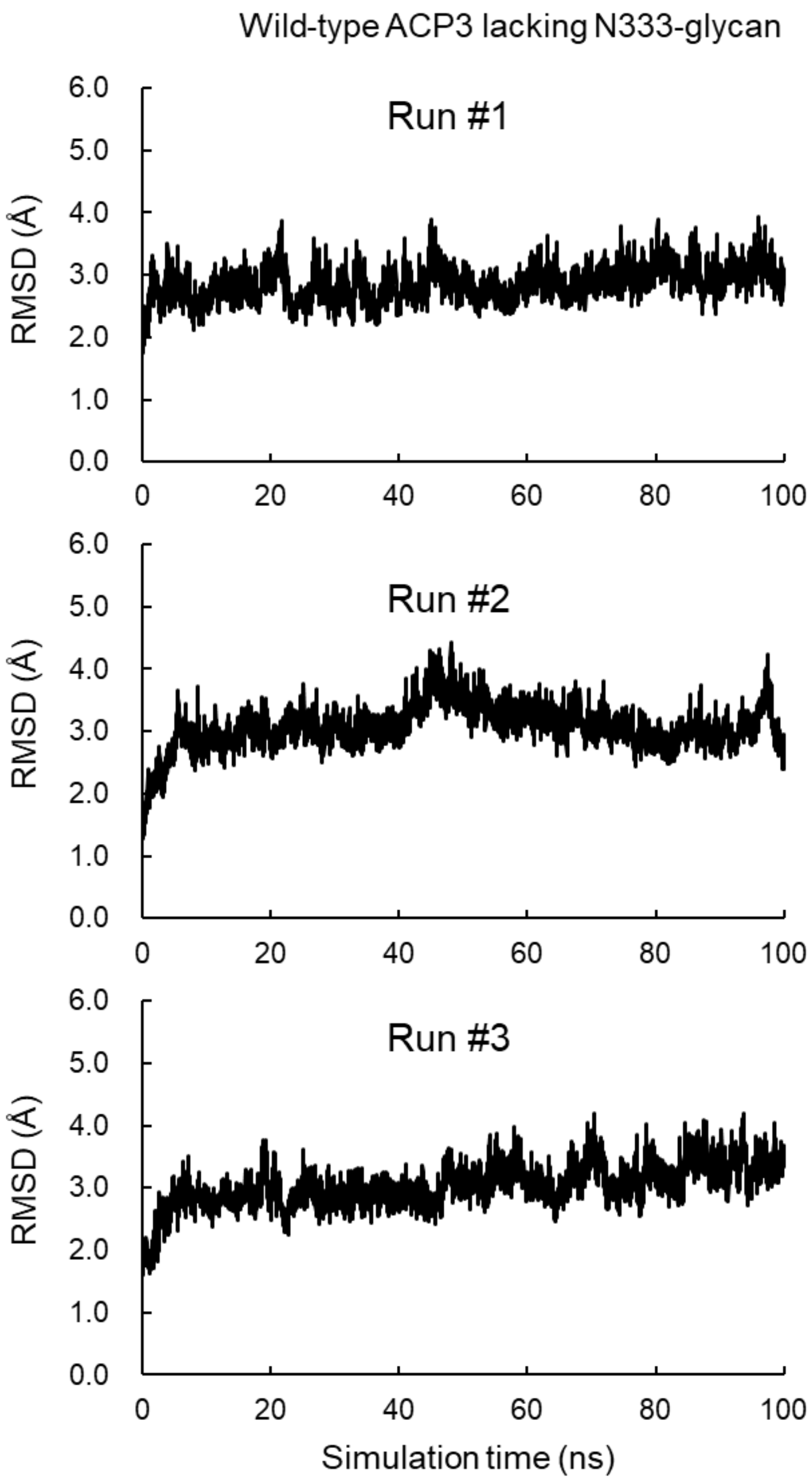

Supplementary Figure 14. Root-mean-square deviation (RMSD) analysis of ACP3 lacking the Asn333 *N*-glycan during MD simulations. RMSD profiles are shown for ACP3 lacking the Asn333 *N*-glycan (n = 3 independent simulations).

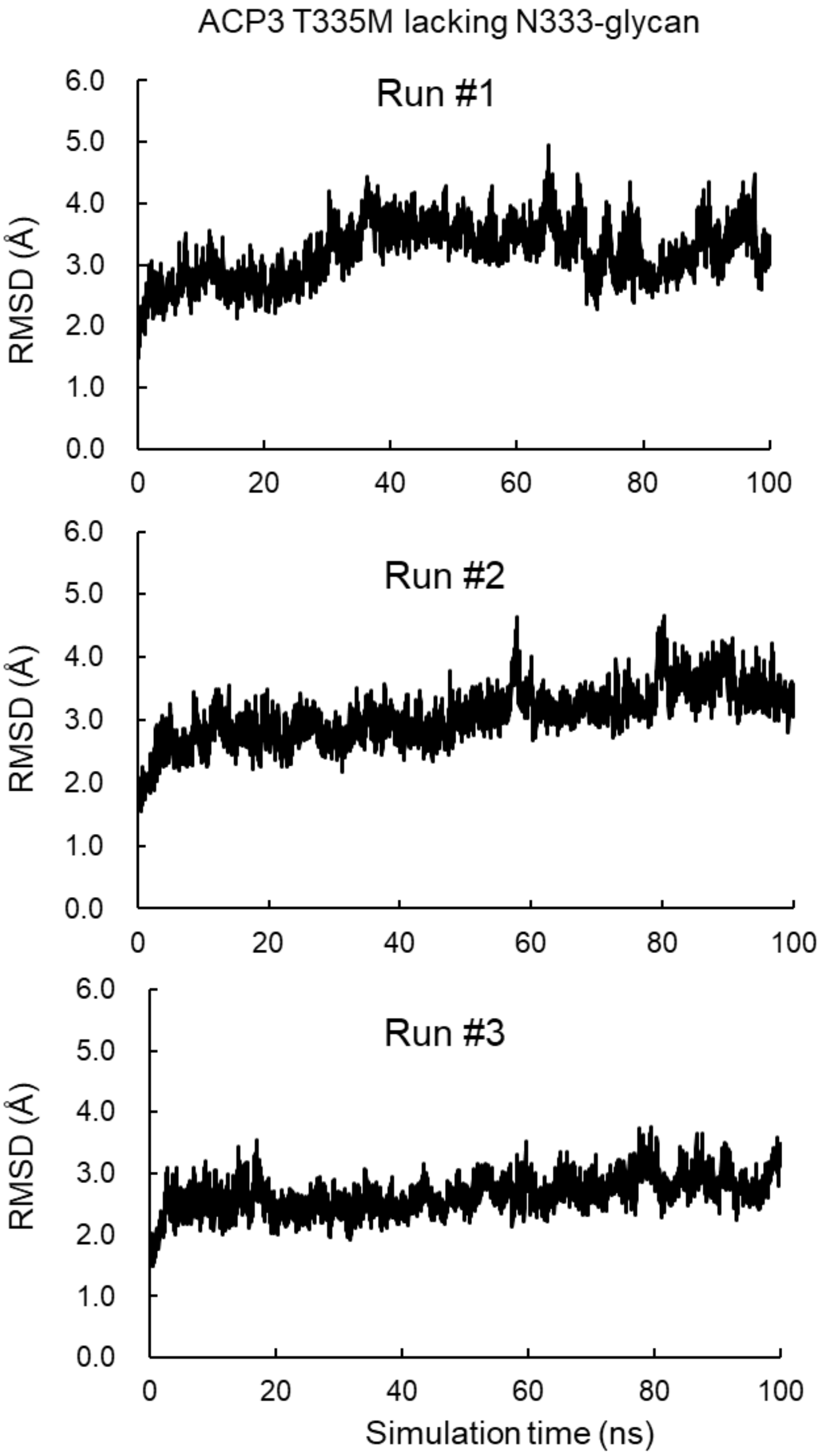

Supplementary Figure 15. Root-mean-square deviation (RMSD) analysis of ACP3 T335M lacking the Asn333 *N*-glycan during MD simulations. RMSD profiles are shown for ACP3 T335M lacking the Asn333 *N*-glycan (n = 3 independent simulations).

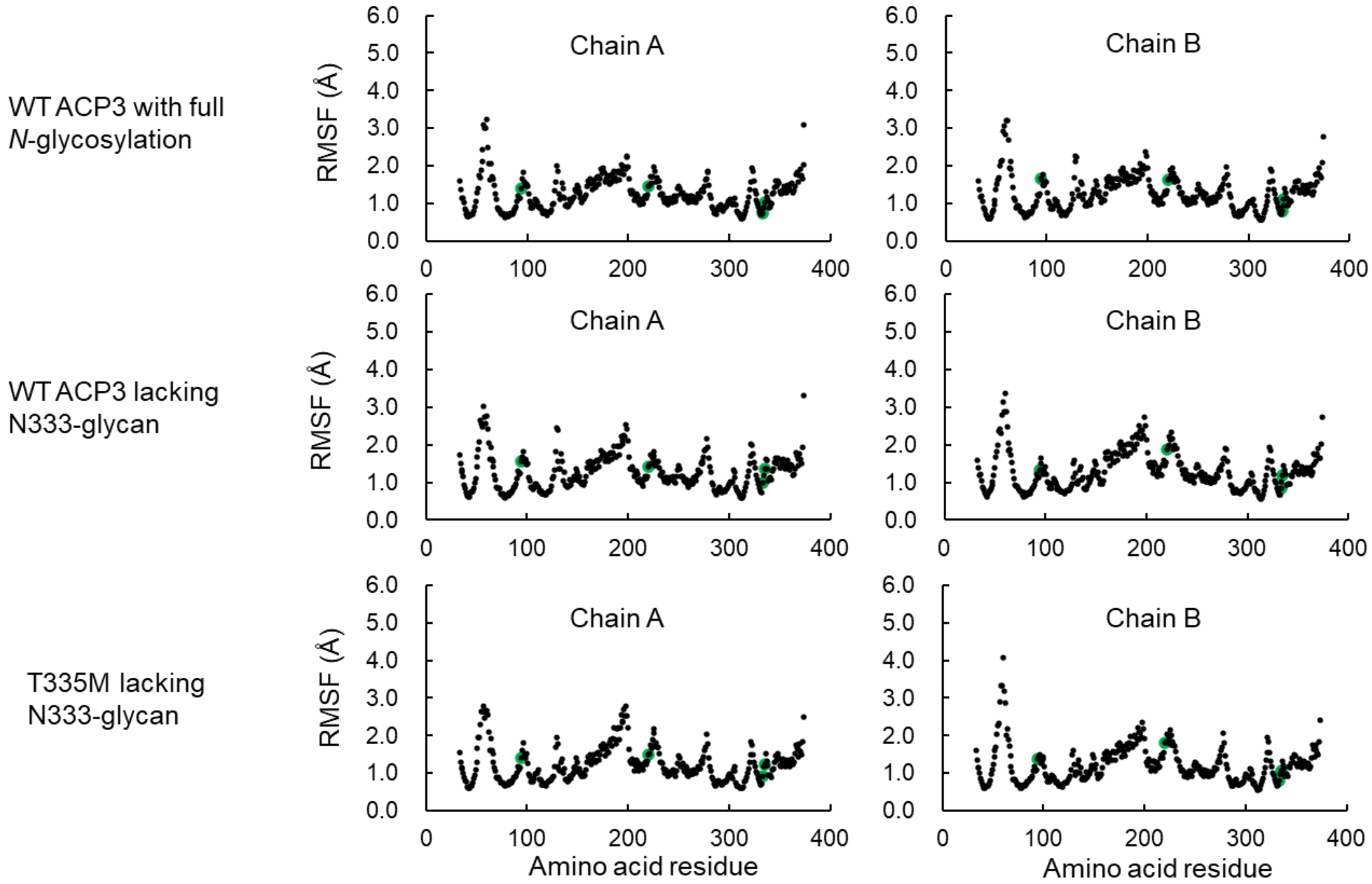

	Asn94		Asn220		Asn333		Thr335/Met335	
	Chain A	Chain B	Chain A	Chain B	Chain A	Chain B	Chain A	Chain B
WT ACP3 with full *N*-glycosylation	1.4 ± 0.1	1.7 ± 0.3	1.5 ± 0.1	1.6 ± 0.2	0.8 ± 0.1	0.8 ± 0.1	1.0 ± 0.1	1.1 ± 0.1
WT ACP3 lacking N333-glycan	1.6 ± 0.0	1.3 ± 0.2	1.4 ± 0.0	1.9 ± 0.3	1.0 ± 0.2	0.8 ± 0.0	1.4 ± 1.1	1.2 ± 0.1
T335M lacking N333-glycan	1.4 ± 0.1	1.4 ± 0.1	1.5 ± 0.1	1.8 ± 0.1	0.9 ± 0.0	0.8 ± 0.0	1.2 ± 0.1	1.1 ± 0.0

Supplementary Figure 16. Root-mean-square fluctuation (RMSF) analysis of ACP3 during MD simulations. RMSF profiles are shown for WT ACP3 with three *N*-glycans, ACP3 lacking the Asn333 *N*-glycan, and ACP3 T335M lacking the Asn333 *N*-glycan. Average RMSF values were calculated from three independent simulations. Average RMSF values for Asn94, Asn220, Asn333 and Thr335/Met335 (green circle) are shown at the bottom with standard deviations.

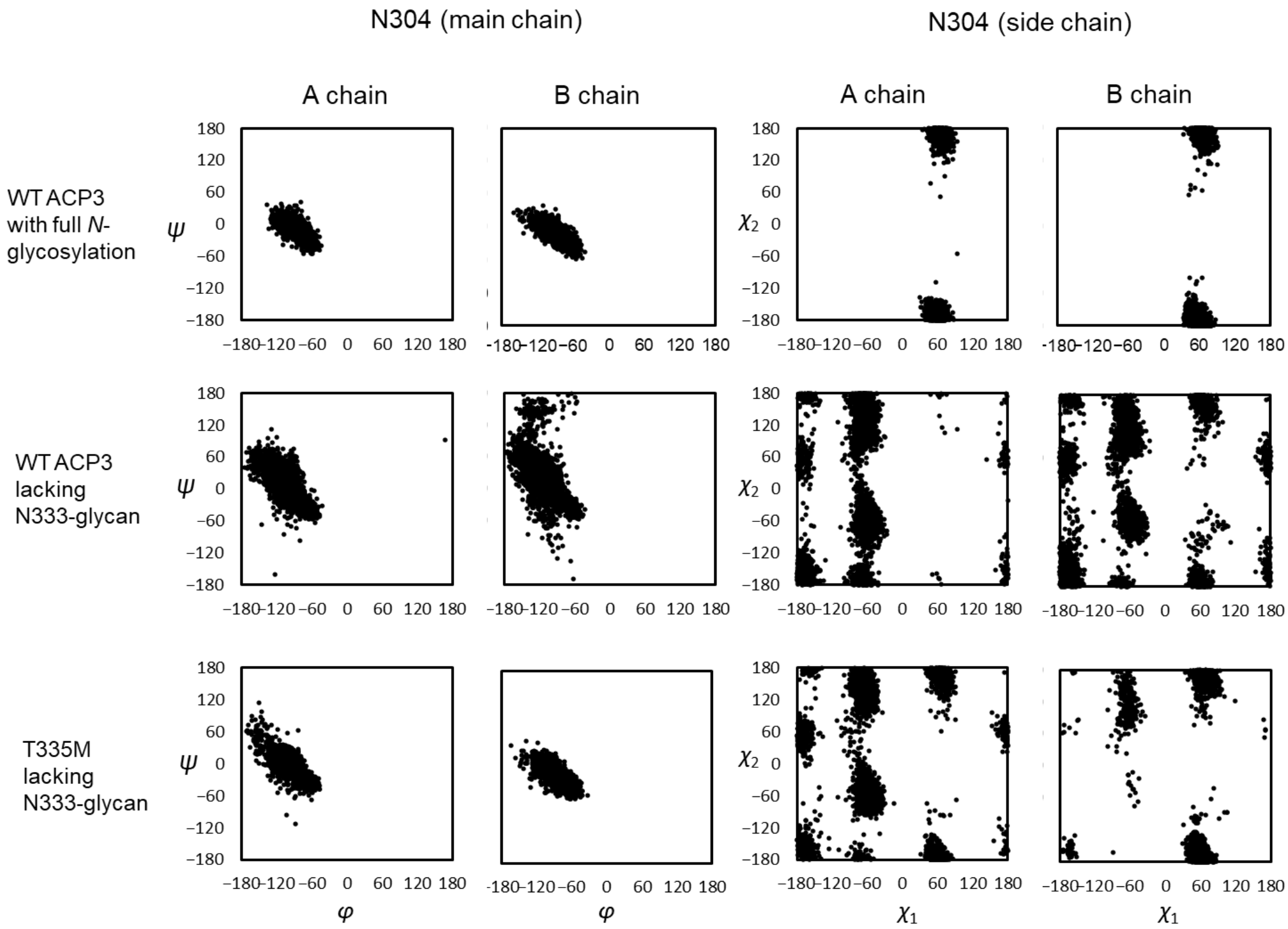

Supplementary Figure 17. Torsion angle distributions of Asn304 during MD simulations. Two-dimensional plots of main chain (left panels) and side-chain (right panels) torsion angles of Asn304 are shown for WT ACP3 carrying three *N*-glycans (top panels), ACP3 lacking the Asn333 *N*-glycan (middle panels), and ACP3 T335M lacking the Asn333 *N*-glycan (bottom panels).

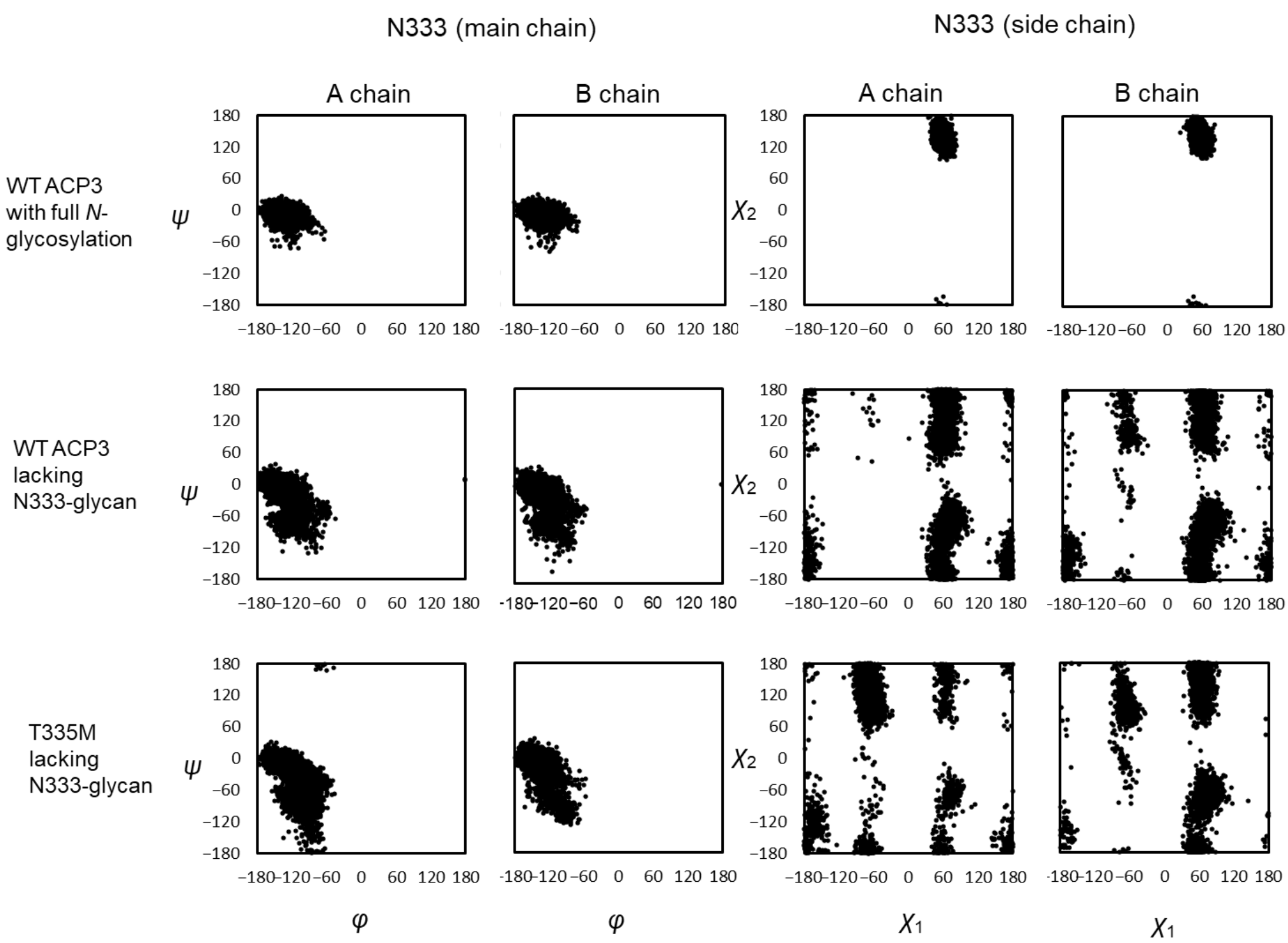

Supplementary Figure 18. Torsion angle distributions of Asn333 during MD simulations. Two-dimensional plots of main chain (left) and side-chain (right) torsion angles of Asn333 are shown for WT ACP3 carrying three *N*-glycans (top panels), ACP3 lacking the Asn333 *N*-glycan (middle panels), and ACP3 T335M lacking the Asn333 *N*-glycan (bottom panels).

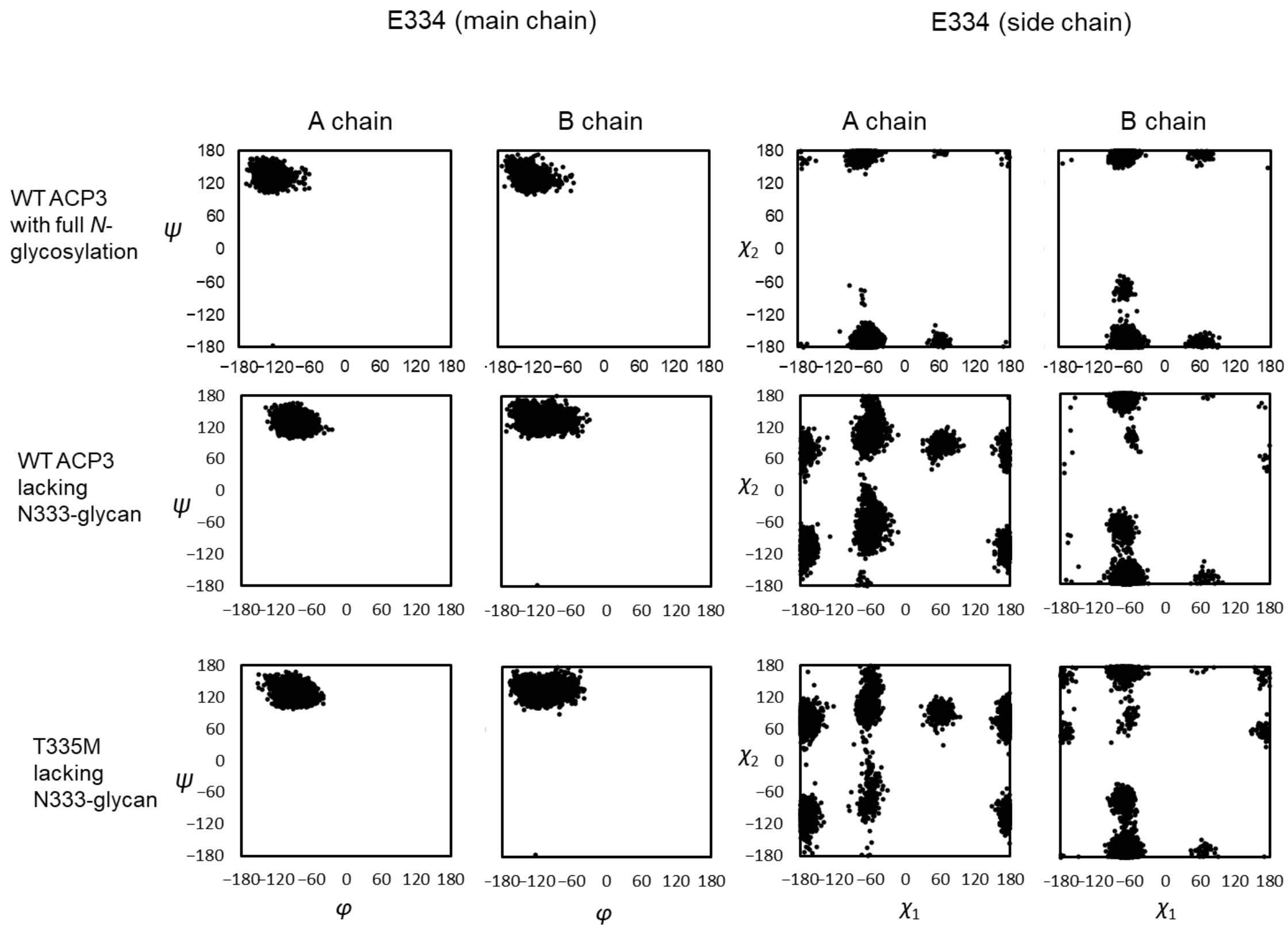

Supplementary Figure 19. Torsion angle distributions of Glu334 during MD simulations. Two-dimensional plots of main chain (left) and side-chain (right) torsion angles of Glu334 are shown for WT ACP3 carrying three *N*-glycans (top panels), ACP3 lacking the Asn333 *N*-glycan (middle panels), and ACP3 T335M lacking the Asn333 *N*-glycan (bottom panels).

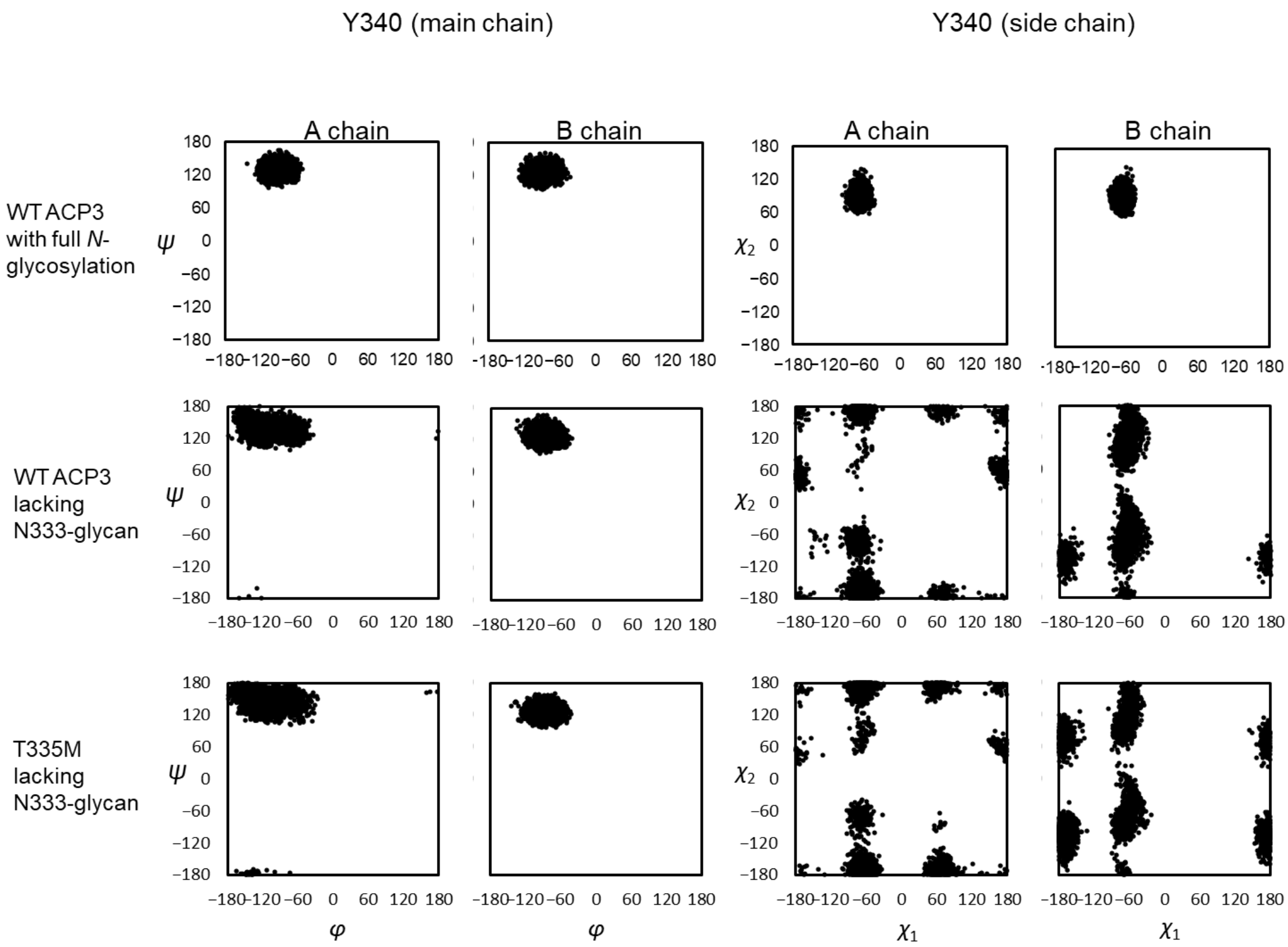

Supplementary Figure 20. Torsion angle distributions of Tyr340 during MD simulations. Two-dimensional plots of main chain (left) and side-chain (right) torsion angles of Tyr340 are shown for WT ACP3 carrying three *N*-glycans (top panels), ACP3 lacking the Asn333 *N*-glycan (middle panels), and ACP3 T335M lacking the Asn333 *N*-glycan (bottom panels).

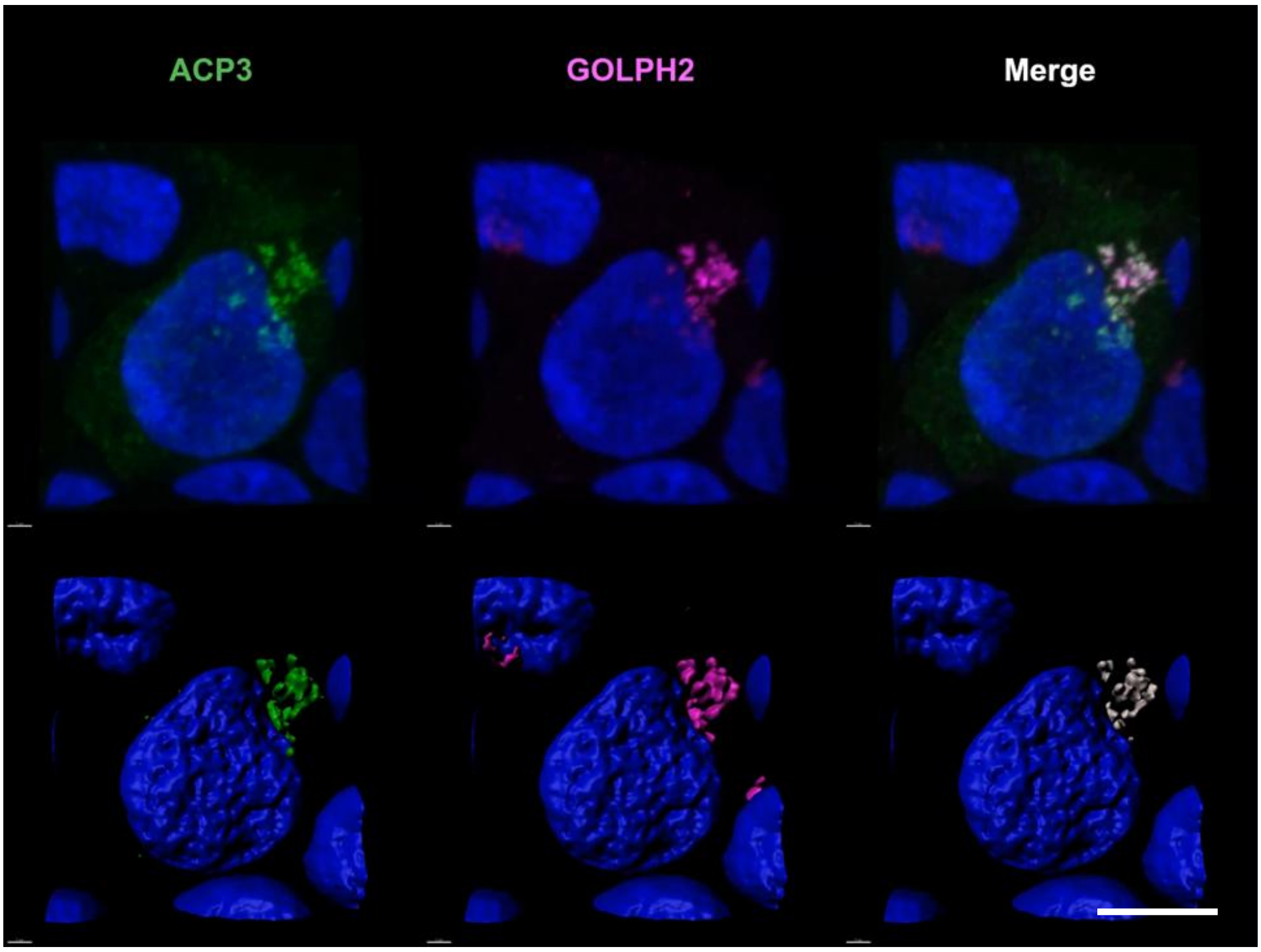

Supplementary Figure 21. Golgi localization of WT ACP3 (Video S1 still). Three-dimensional confocal images and surface-rendered models of HEK293 cells expressing WT ACP3. ACP3 and nuclei are labeled with anti-His tag antibody (green) and Hoechst 33258 (blue), respectively. Colocalization of ACP3 with the Golgi marker GOLPH2 (magenta) is shown. Scale bar, 10 µm.

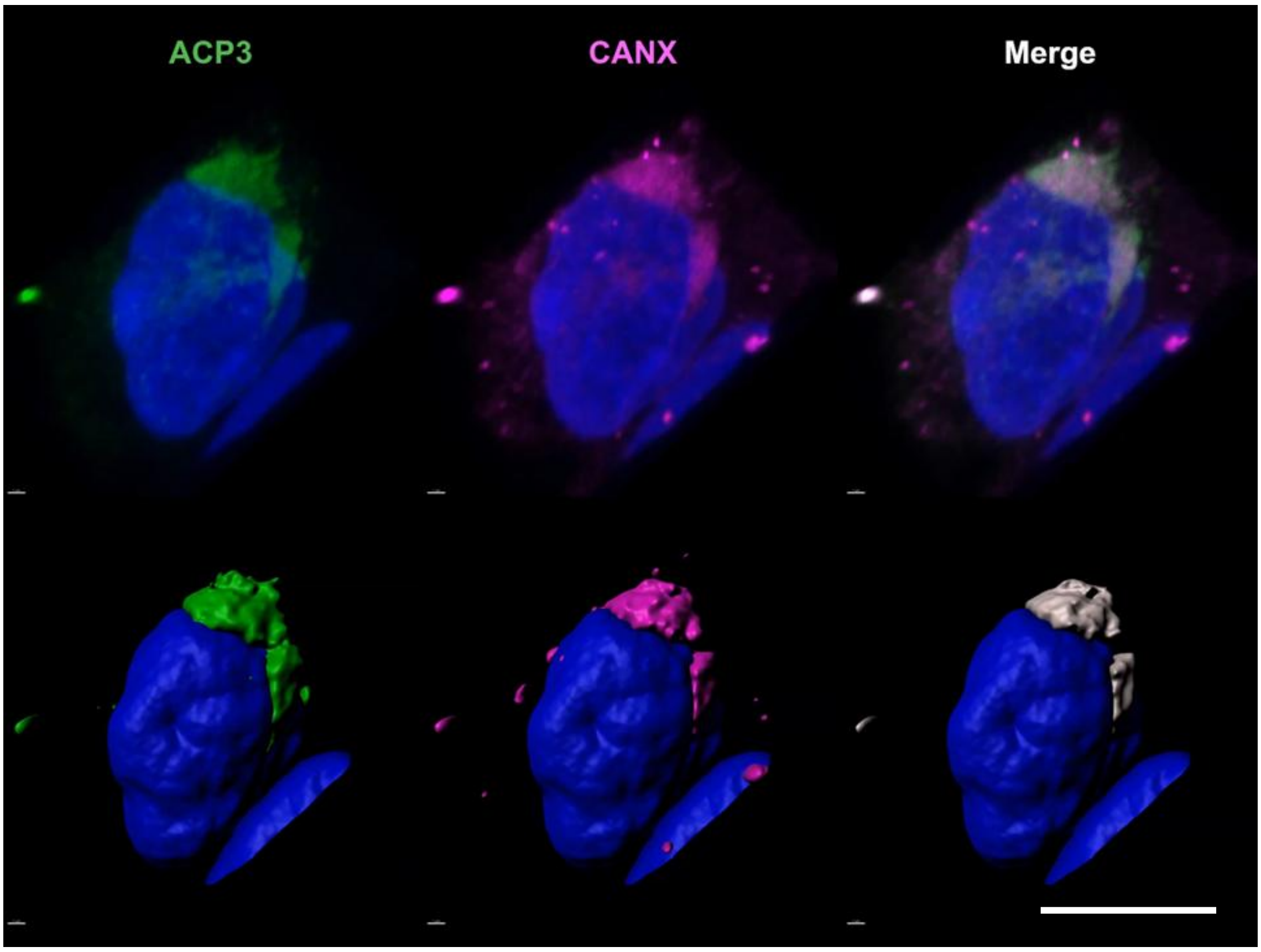

Supplementary Figure 22. Aggregate formation of ACP3 N333Q (Video S2 still). Three-dimensional confocal images and surface-rendered models of HEK293 cells expressing ACP3 N333Q. ACP3 and nuclei are labeled with anti-His tag antibody (green) and Hoechst 33258 (blue), respectively. Colocalization of ACP3 aggregates with the ER marker calnexin (CANX; magenta) is shown. Scale bar, 10 µm.

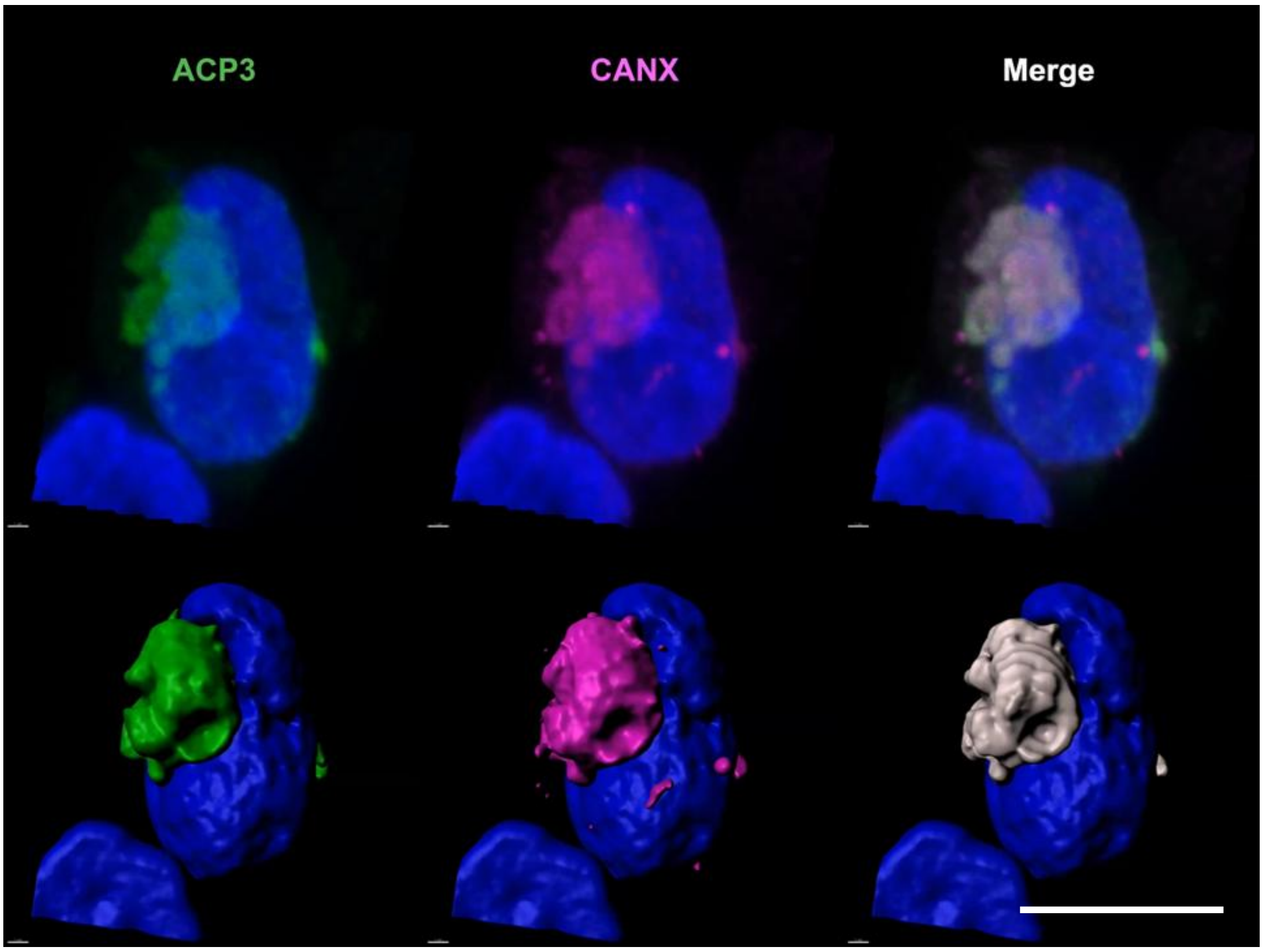

Supplementary Figure 23. Aggregate formation of ACP3 T335M (Video S3 still). Three-dimensional confocal images and surface-rendered models of HEK293 cells expressing ACP3 T335M. ACP3 and nuclei are labeled with anti-His tag antibody (green) and Hoechst 33258 (blue), respectively. Colocalization of ACP3 aggregates with the ER marker calnexin (CANX; magenta) is shown. Scale bar, 10 µm.
